# Histone deacetylase inhibitors inhibit metastasis by restoring a tumor suppressive microRNA-150 in advanced cutaneous T-cell lymphoma

**DOI:** 10.18632/oncotarget.13810

**Published:** 2016-12-07

**Authors:** Fumito Abe, Akihiro Kitadate, Sho Ikeda, Junsuke Yamashita, Hiroki Nakanishi, Naoto Takahashi, Chikara Asaka, Kazuaki Teshima, Tomomitsu Miyagaki, Makoto Sugaya, Hiroyuki Tagawa

**Affiliations:** ^1^ Department of Hematology, Nephrology, and Rheumatology, Akita University Graduate School of Medicine, Akita, Japan; ^2^ Division of Bioscience Center, Radioisotope, Akita University, Akita, Japan; ^3^ Research Center for Biosignal, Akita University, Akita, Japan; ^4^ Department of Otolaryngology, Noshiro Kousei Medical Center, Noshiro, Japan; ^5^ Department of Hematology, Hiraka General Hospital, Yokote, Japan; ^6^ Department of Dermatology, University of Tokyo, Tokyo, Japan

**Keywords:** HDACI, miR-150, CCR6, CTCL, metastasis

## Abstract

Tumor suppressive microRNA (miR)-150 inhibits metastasis by combining with the C-C chemokine receptor 6 (CCR6) “seed sequence” mRNA of the 3′-untranslated region (3′-UTR) in advanced cutaneous T-cell lymphoma (CTCL). Because the histone deacetylase inhibitor (HDACI) vorinostat showed excellent outcomes for treating advanced CTCL, HDACIs may reduce the metastasis of CTCL by targeting miR-150 and/ or CCR6. To examine whether these candidate molecules are essential HDACI targets in advanced CTCL, we used the My-La, HH, and HUT78 CTCL cell lines for functional analysis because we previously demonstrated that their xenografts in NOD/Shi-scid IL-2γnul mice (CTCL mice) induced multiple metastases. We found that pan- HDACIs (vorinostat and panobinostat) inhibited the migration of CTCL cells and downregulated CCR6. The miRNA microarray analysis against CTCL cell lines demonstrated that these pan-HDACIs commonly upregulated 161 miRNAs, including 34 known tumor suppressive miRNAs such as miR-150. Although 35 miRNAs possessing the CCR6 “seed sequence” were included in these 161 miRNAs, miR-150 and miR-185-5p were downregulated in CTCL cells compared to in normal CD4+ T-cells. The transduction of 12 candidate miRNAs against CTCL cells revealed that miR-150 most efficiently inhibited their migration capabilities and downregulated CCR6. Quantitative reverse transcriptase-polymerase chain reaction demonstrated that miR-150 was downregulated in advanced but not early CTCL primary cases. Finally, we injected miR-150 or siCCR6 into CTCL mice and found that mouse survival was significantly prolonged. These results indicate that miR-150 and its target, CCR6, are essential therapeutic targets of pan-HDACIs in advanced CTCL with metastatic potential.

## INTRODUCTION

Cutaneous T-cell lymphoma (CTCL) mainly comprises mycosis fungoides (MF) and Sezary syndrome (SzS) [1–3]. MF is the most common form of CTCL and a good model for understanding the multistep process of cancer development and progression, owing to the clearly defined “early” and “advanced” stages [1–3]. Patients with early-stage MF (patch and plaque) have a good prognosis; however, the advanced stages are associated with a progression to erythroderma and multiple tumors, which are characterized by an aggressive clinical course with shortened survival and multiple metastases [1–3]. Between the early and advanced stages of MF, additional genetic or epigenetic alterations may occur and likely contribute to MF progression and aggressive clinical behavior. These alterations could involve coding or non-coding genes or both including microRNA (miRNA), which are a class of small non-coding regulatory RNA molecules that pair with the 3′-untranslated region (UTR) of target messenger RNAs to repress their translation [4, 5].

The altered expression of various oncogenic and tumor-suppressive miRNAs has been identified in lymphomas/leukemias and solid tumors [6–8]. We previously demonstrated that the expression level of miR-150 was suppressed in both T/NK cell lymphoma [9] and advanced CTCL [10]. Furthermore, the enhanced miRNA expression inhibited metastasis and invasion in a mouse model by targeting the C-C chemokine receptor 6 (CCR6). In this study, we further demonstrated that the interaction between CCR6 and its specific ligand, CCL20, plays an important role in increasing the nutrition-dependent migration in advanced CTCL [11], although the mechanism underlying the downregulation of miR-150 in CTCL is still unclear. Because there is no current report on genomic alteration of the miR-150 region and no mutation is evident in the pre-miRNA-150 gene [11], we suspected that epigenetic mechanisms such as the activation of histone deacetylases might be responsible for repressing miR-150 expression in advanced CTCL.

Histone deacetylases are divided into five sub-classes: Class-I (HDAC1, 2, 3, and 8), Class-IIa (HDAC4, 5, 7, and 9), Class-IIb (HDAC6 and 10), Class-III (SIRT1–7), and Class-IV (HDAC11) [12]. These classes differ in their subcellular localization (Class-I HDACs are nuclear while Class-II enzymes are cytoplasmic) and intracellular targets. HDAC inhibitors (HDACIs) are currently emerging as one of the most promising new classes of drugs for the treatment of specific forms of non-Hodgkin's lymphomas. They are particularly active in T-cell, possibly Hodgkin’s, and indolent B-cell lymphomas. Present, several HDACIs are in clinical trials both as single agents or combined with chemotherapy or other biological agents in malignant lymphoma and other hematological malignancies.

For the treatment of CTCL, suberoylanilide hydroxamic acid (SAHA, vorinostat), a pan-HDACI, has been shown to be clinically effective in advanced CTCL [13, 14] and was first approved by the US Food and Drug Administration (FDA) for the treatment of refractory CTCL. Furthermore, vorinostat is known to induce apoptosis and growth inhibition of CTCL cells [15–18]. We also demonstrated that vorinostat induced cellular senescence in CTCL cells and other non-Hodgkin's lymphoma cell lines [18]. Another pan-HDACI, panobinostat, also has been recently approved by the FDA for refractory multiple myeloma [19]. Panobinostat may be effective for refractory CTCL and is currently being evaluated in an ongoing clinical study for peripheral T-cell lymphoma [20]. The Class I-specific HDACI, romidepsin, is also approved for the treatment of refractory CTCL [21] and is being investigated in ongoing clinical trials for other peripheral T-cell lymphomas [22].

Refractory CTCL, especially stage IV cases, recurrently shows invasion and metastasis in multiple visceral organs with upregulation of CCR6 [10, 23]. Increased expression levels of CCR6 have also been reported in various solid cancers, which is closely associated with poor prognosis in these patients [24–27]. Because the recent use of HDACIs in advanced CTCL could contribute to excellent outcomes, we hypothesized that HDACIs might inhibit the migration and reduce the CCR6 expression [13–17, 19–22]. We supposed that HDACIs likely have a key role in restoring the critical tumor suppressive factors and, therefore, it was tempting to consider that HDACIs might induce not only CCR6 reduction but also restoration of tumor suppressive miRNAs. Therefore, in this study, we focused on investigating the relationship between miRNAs and HDACIs, especially by determining the CCR6-associated miRNAs.

## RESULTS

### Vorinostat and panobinostat downregulate CCR6 expression and inhibit migration of CTCL cell lines

**Figure 1:**
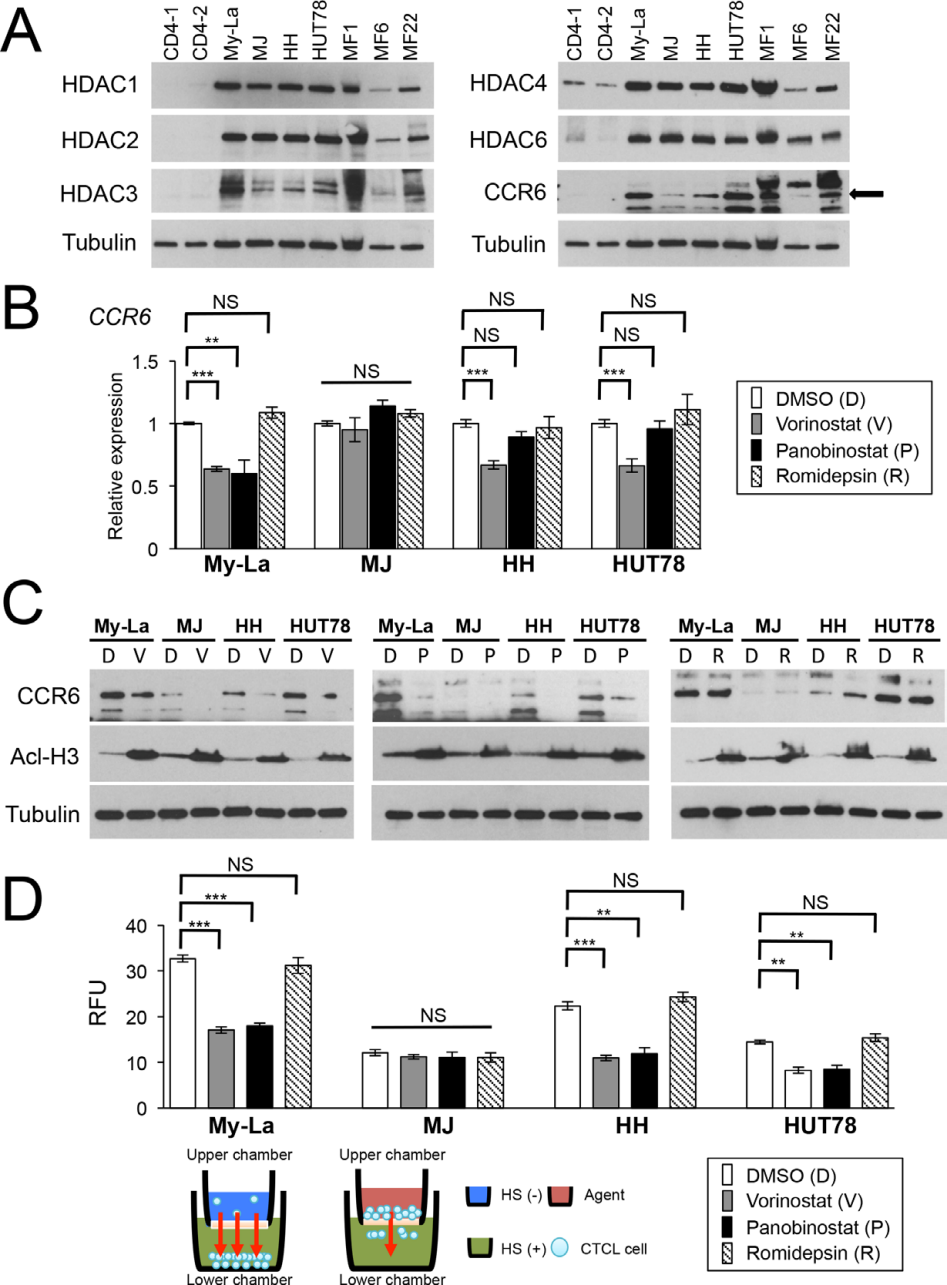
Vorinostat and panobinostat restore expression of CCR6, which inhibits migration of CTCL cells (**A**) Western blot analysis of histone deacetylase (HDAC) 1, 2, 3, 4, and 6 and CCR6 in CTCL cell lines and three cases of primary and advanced CTCL, which were obtained from lymph nodes of patients with MF tumors (namely MF1, MF6, and MF22). Tubulin, control. (**B)** qRT-PCR analysis of CCR6 in HDACIs [vorinostat, panobinostat, Romidepsin-treated CTCL cells (My-La, MJ, HH, and HUT78 cell lines). Student's *t*-test was used to examine significance. Bars are means ± standard error of the mean (SEM) of three independent experiments. **0.001 ≤ *P* < 0.01, ***P < 0.001. NS: not significant. (C) Western blot analysis of CCR6 and acetyl-histone H3 (Acl-H3) in My-La, MJ, HH, and HUT78 cells treated with vorinostat (V), panobinostat (P), and romidepsin (R). DMSO is shown as “D”. Tubulin, control. (**D**) Migration of My-La, MJ, HH, and HUT78 cells treated with vorinostat (5 μM) or panobinostat (80 nM) and romidepsin (10 nM). RFU was measured 16 hr after drug treatment. Student's *t*-test was used to examine significance. Bars are means ± SEM of three independent experiments. Illustration of migration assay is also under bar graph. **0.001 ≤ *P* < 0.01, ***P < 0.001. NS: not significant.qRT-PCR, quantitative reverse transcriptase-polymerase chain reaction; RFU, relative fluorescence units.

To examine HDACs expression, we conducted western blot analysis of HDACs in normal CD4+ T-cells, CTCL cell lines (CD4+My-La, HH, MJ, and HUT78), and three primary CTCL tissue samples (MF1, MF6, and MF22). Tissue samples were obtained from the metastatic lymph nodes of primary cases. Among the four CTCL cell lines, we previously demonstrated that the subcutaneous injection of My-La, HH, and HUT78 into NOD/Shi-scid IL-2γnul mice induced multiple metastases in various visceral organs [10, 11, 18]. Here, we confirmed there was an increased expression of HDACs (1, 2, 3, 4, and 6) and CCR6 in the CTCL cell lines and primary tissue samples (Figure 1A).

To determine whether the HDACIs [pan-HDACIs (vorinostat, panobinostat) and Class I-specific HDACI, romidepsin] affected the expression of CCR6, we examined the expression of *CCR6* by qRT-PCR and western blotting analyses of CTCL cell lines treated with the respective HDACI. We found that vorinostat and panobinostat significantly reduced the expression of *CCR6* (My-La, Figure 1B) and/or its product (My-La, HH and HUT78, Figure 1C), although these pan-HDACIs did not affect the *CCR6*/CCR6 expression level in MJ. In addition, romidepsin did not affect *CCR6*/CCR6 expression in all four CTCL cell lines.

To determine whether HDACIs inhibit the migratory activity of CTCL cells, we performed an *in vitro* migration assay and found that the migration of My-La, HH, and HUT78, but not MJ, cells were inhibited by vorinostat or panobinostat but not by romidepsin treatment (Figure 1D). These results suggest that 1) these pan-HDACIs effectively reduce the migration of CTCL cells by reducing CCR6 expression and 2) pan-HDACIs treatment inhibits miRNA-mediated translational inhibition. In subsequent experiments, we focused on the effect of vorinostat and panobinostat on miRNAs involved in inhibiting the metastatic ability of CTCL cells.

### Pan-HDACIs upregulate various tumor suppressive miRNAs including miR-150 in CTCL cells

**Figure 2:**
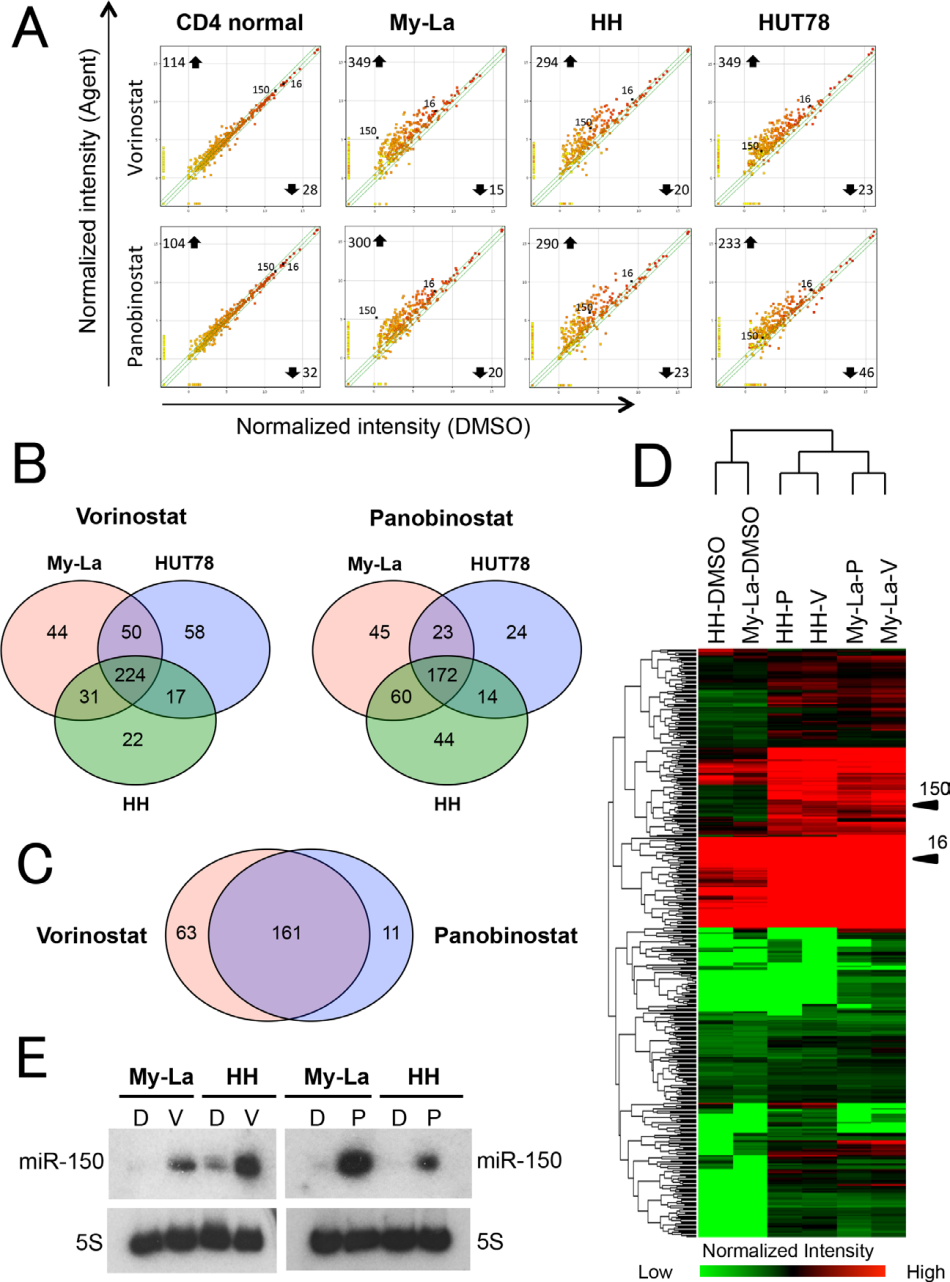
miRNA expression analysis of HDACIs-treated CTCL cell lines (**A)** miRNA expression analysis of CTCL cells and normal CD4+T-cells. X- and Y-axes, intensity of miRNAs treated with dimethyl sulfoxide (DMSO) and pan-HDACIs (vorinostat and panobinostat), respectively. Normal CD4+T-cells, My-La, MJ, HH, and HUT78 cells treated with vorinostat (5 μM) or panobinostat (80 nM) for 24 hr. (**B)** Commonly upregulated miRNAs in My-La, HH, and HUT78 cells treated with vorinostat (left) or panobinostat (right). (**C)** Commonly upregulated miRNAs in My-La, HH, and HUT78 treated with vorinostat and panobinostat. (**D)** Heat map of My-La and HH cell lines treated with and without pan-HDACIs [vorinostat (V), panobinostat (P)]. (**E)** Northern blot analysis of miR-150 in the My-La and HH cell lines treated with vorinostat (V) or panobinostat (P). DMSO (D), control. 5S tRNA (5S), control.

We demonstrated that the downregulation of miR-150 induced metastasis of CTCL cells [10]. Therefore, vorinostat and panobinostat might upregulate tumor suppressive miRNAs including miR-150, leading to migration inhibition. To investigate the effect of vorinostat and panobinostat on miRNA expressions, we examined a miRNA array against My-La, HH and HUT78 cells treated with and without the HDACIs. Here, we defined “upregulation” as a threshold fold change > 1.5 degree higher than the control (dimethyl sulfoxide, DMSO-treated CTCL cell line) miRNAs. Similarly, “downregulation” was defined as a threshold fold change > 1.5 degree lower than the control. When we conducted the miRNA array analysis against the normal CD4+ T-cells that were exposed to vorinostat or panobinostat, we detected significantly lower miRNA expression changes than those of the tumor cells (Figure 2A). Vorinostat commonly upregulated 224 miRNAs and panobinostat upregulated 172 miRNAs in the three CTCL lines (Figure 2B). Among them, 161 miRNAs were commonly upregulated by vorinostat and panobinostat in My-La, HH, and HUT78 cells (Figure 2C). The commonly upregulated miRNAs included those of 34 known tumor suppressor genes (e.g., miR-16, miR-96, miR-150, miR-183, miR-186, miR-194, miR-320, and miR-371), nine miRNAs of oncogenes (e.g. miR-454), and 14 miRNAs that show both tumor suppressive and oncogenic function(Supplementary Table S1). Expectedly, miR-150 was one of the commonly upregulated miRNA. Another interesting finding was that the vorinostat- and panobinostat-exposed HH and My-La cells showed very similar miRNA expressions (Figure 2D) and the upregulation of miR-150 in these cells was also confirmed by the northern blot analysis (Figure 2E).

### Pan-HDACIs upregulate miRNAs that potentially regulate CCR6 in CTCL cells

**Figure 3:**
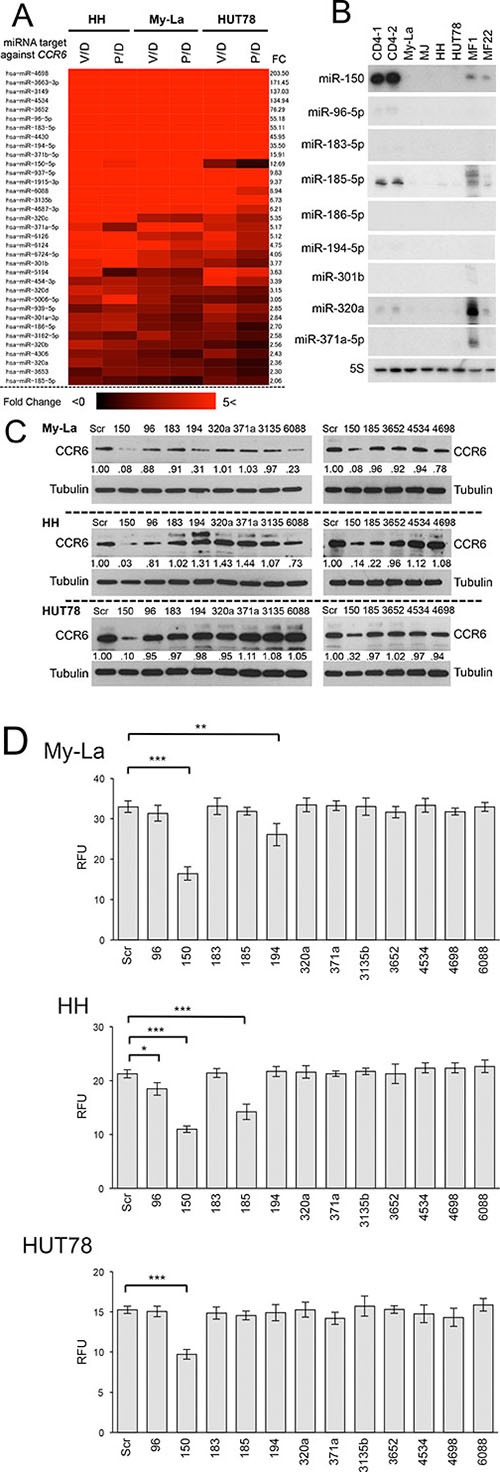
Upregulated miRNAs by HDACIs that potentially inhibit CCR6 in CTCL cells (**A)** Heat map and intensity of miRNAs with seed sequence of CCR6 [FC, fold change: > 1.5]. V/D = vorinostat/DMSO, P/D = panobinostat/DMSO. (**B)** Northern blot analysis of candidate miRNAs with CCR6 seed sequence (miR-96-5p, miR-150, miR-183-5p, miR-194-5p, miR-301b, miR-320c, and miR-371b-5p) showing high fold change (FC: > 1.5) for normal CD4+ cells (CD4-1, and CD4-2), CTCL cell lines, and primary MF samples (*n* = 2, tumor phase). 5S tRNA (5S), control. (**C)** Western blot analysis of CCR6 expression in My-La, HH, and HUT78 cell lines transiently transduced with respective candidate miRNAs (Scr: scrambled; 96: miR-96-5p; 150: miR-150; 183: miR-183-5p; 185: miR-185-5p; 194: miR-194-5p; 301: miR-320a; 371: miR-371a-5p; 3135: miR-3135b; 3652: miR-3652; 4534: miR-4534; 4698: miR-4698; 6088: miR-6088). Tubulin, control. (**D)** Migration assay using miR-96-5p, miR-150, miR-183-5p, miR-185-5p, miR-194-5p, miR-320a, miR-371a-5p, miR-3135b, miR-3652, miR-4534, miR-4698, miR-6088 and scrambled control (Scr) against My-La, HH, and HUT78 cells. RFUs (relative fluorescence units) were measured 16 hr after drug treatment. Student's *t* test was used for examining significance. Bars are means ± standard error of the mean (SEM) of three independent experiments. *0.01 ≤ *P* < 0.05, **0.001 ≤ *P* < 0.01, ***P < 0.001. Student's *t*-test was used to examine significance.

It is known that miRNA can inhibit the RNA-protein translation by combining with the seed sequence of 3′UTR of the target gene [4, 5]. The seed sequence comprising 7–8 nucleotides is usually in the untranslated region of the target mRNA, and has been conserved in vertebrates and mammals [4, 5]. Among 161 miRNAs that were commonly upregulated in My-La, HH and HUT78 cells with HDACIs, 35 miRNAs possessed seed sequence of CCR6 (Figure 3A). These miRNAs have a potential to directly downregulate CCR6.

To examine the miRNAs that has a functional role in CTCL metastasis, we conducted northern blot analysis of these candidate miRNAs in the CTCL cell lines and primary tissue samples (Figure 3B). We conducted the northern blot using 27 probes of the 35 candidates. Among the 35 probes, 12 (such as the miR-6088) showed unspecific hybridization to the ribosomal RNAs, and therefore, we did not evaluate those results. This unspecific hybridization might have been because the sequence of these probes lacked sufficient specificity to interact with target miRNA (Supplementary Table S2). We found that the miR-150, at least, was strongly expressed in normal CD4^+^ cells, but the others showed very low or no expressions. This result suggests that only the miR-150 might be functional and tumor significant in CTCL metastasis. In addition, to examine whether these candidate miRNAs regulate CCR6, we transiently transduced 12 miRNAs including miR-150, miR-96, miR-183, miR-194-5p, miR-320a, miR-371a-5p, miR-3135b, miR-3652, miR-4534, miR-4698, and miR-6088. The western blot analysis demonstrated that CCR6 was commonly downregulated by the transduction of miR-150 in the My-La, HH, and HUT78 cells (Figure 3C). A migration assay using these miRNAs was further conducted in the My-La, HH, and HUT78 cells, which revealed that miR-150 inhibited the migration of these three cell lines (Figure 3D). These data strongly suggest that the most likely target miRNA of the pan-HDACIs in metastasis inhibition was the miR-150 in the metastatic CTCL.

### Vorinostat restores miR-150 and inhibits migration independently of p53 status

**Figure 4:**
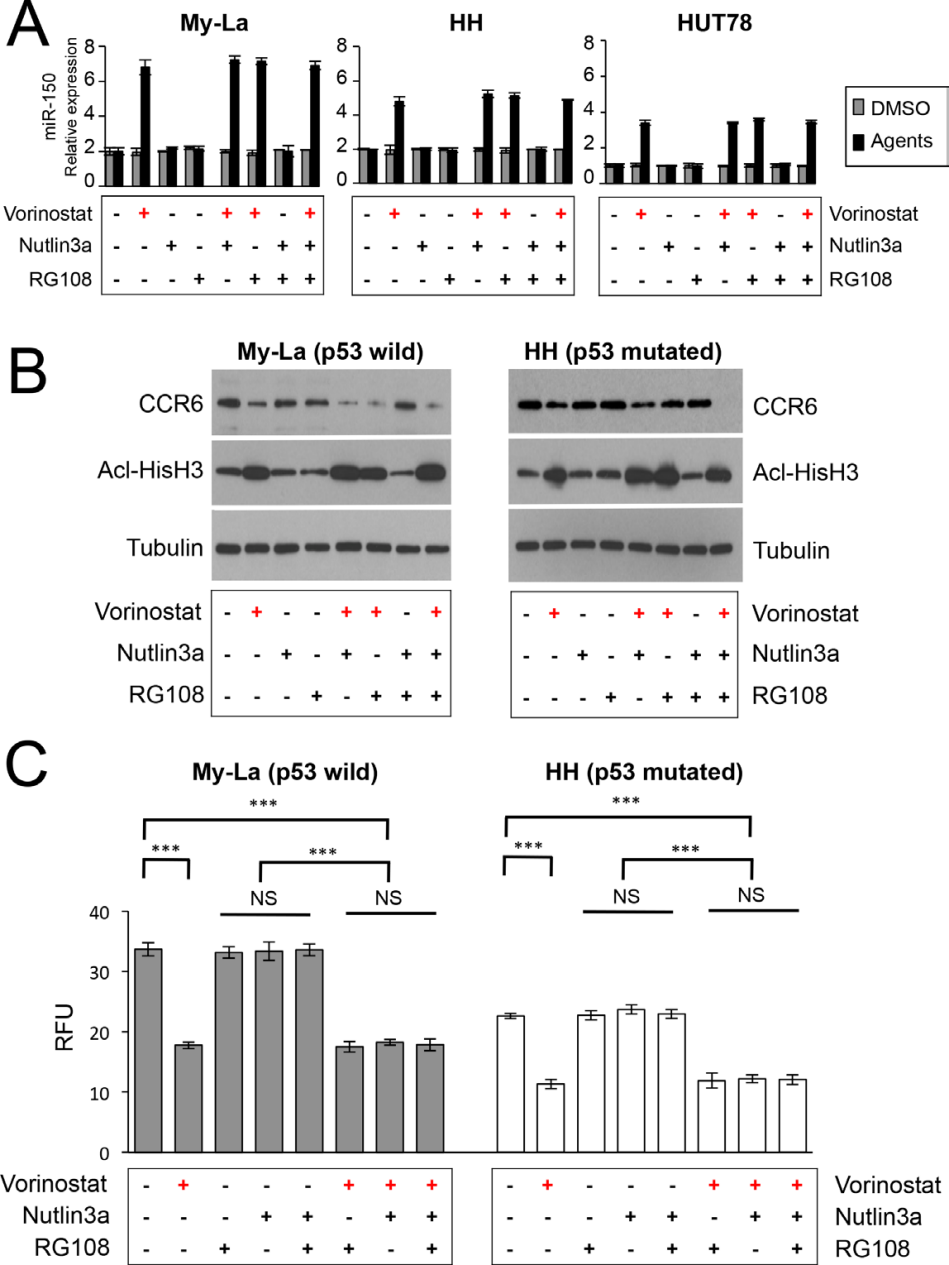
Examination of combined effects of vorinostat, RG108, and nutlin-3a against CTCL cells (**A)** Expression of miR-150 in My-La, HH, and HUT78 cells treated with histone deacetylase inhibitor (HDACI, vorinostat, 5.0 μM), MDM2 inhibitor [nutlin-3a, 2.5 μM (My-La), 20 μM (HH and HUT78)], and/or methyltransferase inhibitor (RG108, 200 μM). ” + “: treatment of agent [vorinostat (red), nutlin3a (black), RG108 (black)] , “ – “: no treatment of agent. (**B)** Western blot analysis of C-C chemokine receptor 6 (CCR6) and acetyl-histone H3 (Acl-HisH3) in My-La and HH cells with treated with HDACI (vorinostat, 5.0 μM), MDM2 inhibitor [nutlin-3a, 2.5 μM (My-La), 20 μM (HH)] and/or methyltransferase inhibitor (RG108, 200 μM). Tubulin, control. (**C)** Migration assay of My-La and HH cells with treated with HDACI (vorinostat, 5.0 μM), MDM2 inhibitor [nutlin-3a, 2.5 μM (My-La), 20 μM (HH)] and/or methyltransferase inhibitor (RG108, 200 μM). Student's *t*-test was used to examine significance. Bars are means ± standard error of the mean (SEM) of three independent experiments. ***P < 0.001. NS: not significant.

In recent our study, we demonstrated that miR-16 was repressed in early to advanced CTCL. Along with inhibition of cell proliferation, miR-16 induced apoptosis or cellular senescence [18]. In this study, we also demonstrated that the induction of apoptosis or senescence was dependent on p53 status: miR-16 induced apoptosis in p53-mutated CTCL, but senescence in the p53 wild-type. Based on this finding, we determined whether miR-150 expression was also affected by p53 status by examining the combination effect of vorinostat and nutlin-3a [an inhibitor of MDM2, an activation factor of p53 (refs. 28, 29)] on miRNA expression. My-La expresses wild type p53 and carries the wild-type TP53 gene, whereas HH does not express p53 because of a splicing alteration (HH) in TP53 [30]. Among the agents investigated, we found that only vorinostat dose-dependently increased the expression of miR-150 in the My-La and HH cells (Figure 4A). Nutlin-3a had no effect on the expression of CCR6 alone or in combination with vorinostat (Figure 4B). Moreover, because nutlin-3a did not enhance the migration inhibitory effect of vorinostat any further in the TP53 wild-type, My-La and TP53 mutated HH cells (Figure 4C), the migration inhibition by vorinostat could be independent of p53 function. In addition, a previous study showed that miR-150 silencing occurred through a DNA-methylation mechanism in anaplastic large cell lymphomas [31]. To examine whether this methylation mechanism for miR-150 downregulation also occurs in CTCL, we examined the effects of a methyltransferase inhibitor (RG108) on CTCL cells. However, RG108 in My-La and HH cells could not restore the expression of miR-150 (Figure 4A–4C), suggesting that miR-150-silencing by DNA methylation does not occur in CTCL. These results also support our hypothesis that miR-150 is downregulated by HDAC activation and that the inhibition of migration by miR-150 occurred independently of the p53 status in CTCL.

### Downregulation of miR-150 in advanced CTCL

**Figure 5:**
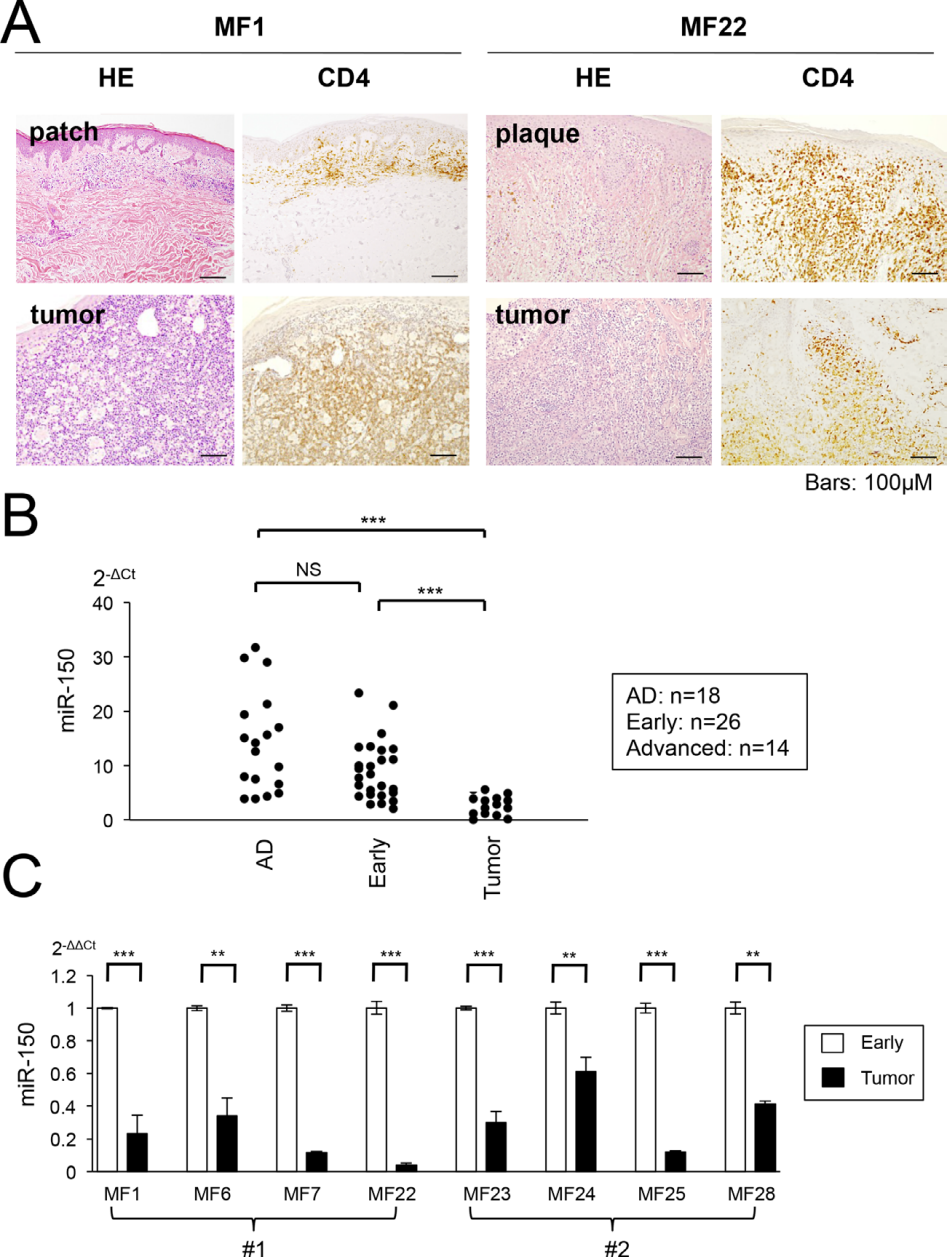
miR-150 is downregulated during progression of primary CTCL cases (**A**) Photomicrographs of early and tumor phase of mycosis fungoides (MF) samples collected from same patients (MF1 and MF22). Hematoxylin and eosin (H&E) and CD4+ staining are shown. (**B)** qRT-PCR analysis of miR-150 in tissue samples of normal atopic dermatitis (AD, *n* = 18), early MF (*n* = 26), and tumor phase MF (*n* =14). Y-axis: 2-ΔCt values for miRNA expression. P-values were calculated using Mann-Whitney *U* test. ***P < 0.001. NS: not significant. (**C)** Comparison of miR-150 expression between early and tumor MF in same patients (eight cases). #1: miR-150 expression of different clinical courses (MF1, MF6, MF7, and MF22), #2: miR-150 expression of the same patient from different biopsy regions patch/plaque and tumor (MF23, MF24, MF25, and MF28). Student's *t*-test was used to examine significance. **0.001 ≤ *P* < 0.01, ***P < 0.001. Bars are means ± standard error of the mean (SEM) of three independent experiments.

The results presented in Figure 3 strongly suggest that miR-150 might be epigenetically silenced during the progression of CTCL. The suppression of miR-150 appears to be one of the genetic or epigenetic events that might occur in the late stage of CTCL and underlies the invasiveness/metastasis of CTCL cells. Although we showed that miR-150 was downregulated significantly in the lymph-node tissue samples from advanced CTCL [10], its expression in the early stage has not been examined. Therefore, to examine the expression of miR-150 in primary CTCL, we conducted a qRT-PCR analysis of samples from early and advanced cases. Patients were classified into early cases (patch and/or plaque MF) and advanced cases (tumor MF) according to their types of skin lesions and 32 cases were enrolled (Figure 5A, the detailed sample information is described in Supplementary Table S3). Among these, eight cases showed both early and advanced phases as follows; four cases exhibited simultaneous patch/plaque and tumor legions in the same patient at diagnosis while the other four cases showed disease progression during the clinical course of each patient. We obtained 26 samples with “patch, plaque, or both” and 14 “tumor” specimens. The control samples were obtained from patients with atopic dermatitis (AD, *n* = 18). It is noteworthy that a micro-dissection was conducted to obtain the “CD4^+^ region” but not the CD4^+^ “cell.” Furthermore, the majority of dissected regions might be normal or tumor CD4^+^ cells, but the sample may contain a small population of other cells or tissue.

The qRT-PCR demonstrated that the expression of miR-150 was significantly lower in the advanced specimens than it was in samples from patients with AD. The miR-150 levels did not significantly differ between the early and the AD specimens, but a significant difference was detected between the early and advanced specimens (Figure 5B). We compared the miR-150 expression of six individual cases, which were samples from the same patients [four cases each were samples from different clinical time points (#1) and different legions of the same time course (#2)]. We demonstrated that the miR-150 expression level was significantly higher in the early specimen than it was in the advanced specimen, although there was no significant difference in the expression between AD and the early samples. In addition, although we conducted a qRT-PCR analysis of miR-185-5p in the same cohort, we did not detect any significant downregulation (data not shown). These results suggest that miR-150 expression declines with disease progression in CTCL (Figure 5C). Moreover, its expression is different between early lesions (patch/plaque) and advanced lesions (tumor) even in the same patient.

### *In vivo* administration of miR-150 to CTCL-xenografted mice prolonged their survival

**Figure 6:**
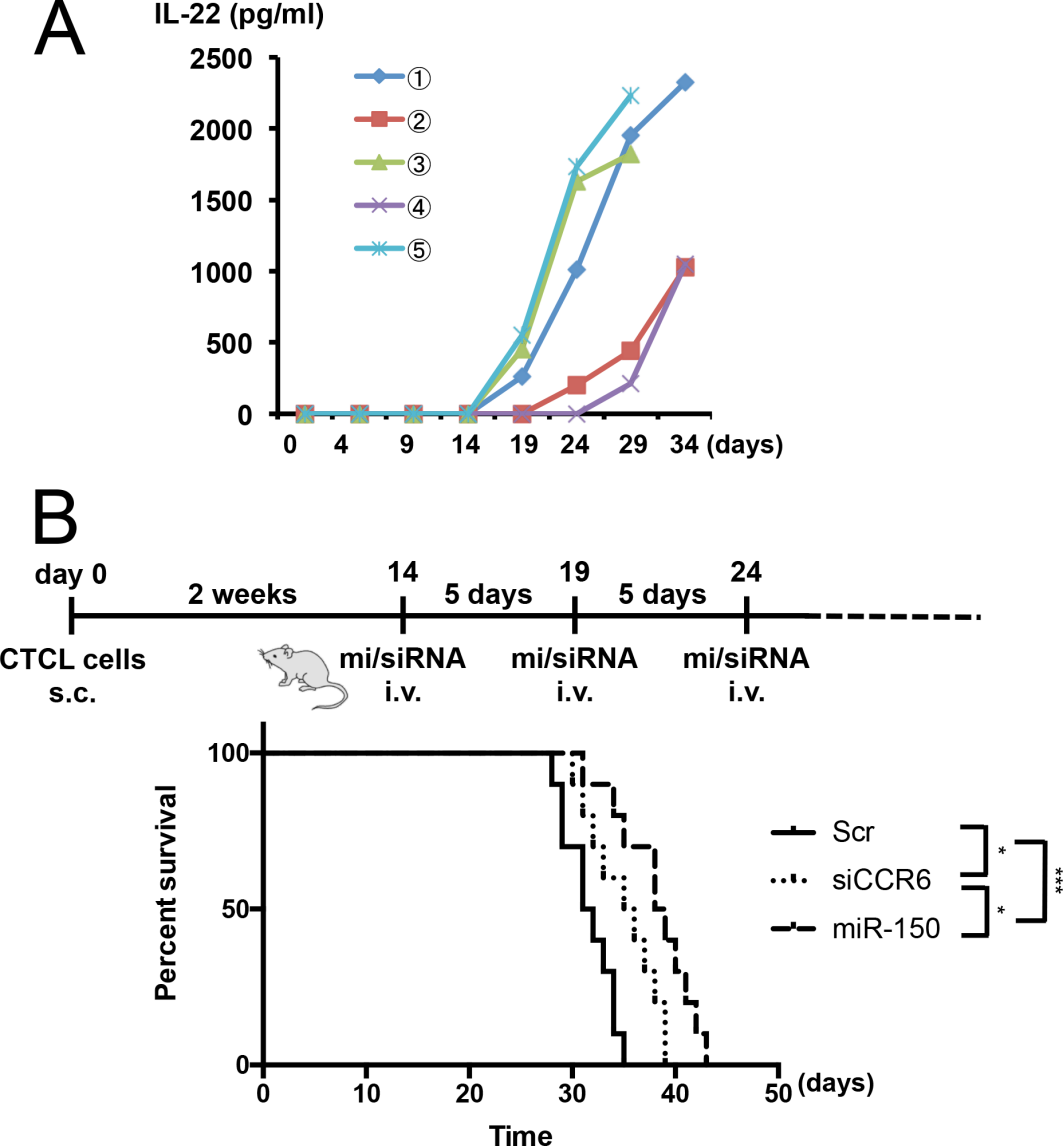
*In vivo* administration of miR-150 to CTCL xenograft mouse model Schematic diagram of injection protocol. (**A)** ELISA (enzyme-linked immunosorbent assay) of IL-22 in serum of NOG mice transplanted with My-La. Serum IL-22 levels in NOG mice transplanted with My-La cells. Blood samples were collected from tail vein every 5 days after the transplantation. X-axis: day after My-La cells’ transplantation for NOG mice. Y-axis: concentration of IL-22 (pg/mL). (**B)** Kaplan-Meier survival curves for My-La mice administered scrambled-control (30 μM, *n* = 10), miR-150 (30 μM, *n* = 10), or si (small interfering) CCR6 (30 μM, *n* = 10)]. s.c.: subcutaneous injection (2 × 105 cells). i.v.: intravenous injection. Log-lank test was conducted for statistical analysis. *0.01 ≤ *P* < 0.05, ***P < 0.001.

We previously established a mouse model of metastasis and invasion of CTCL in NOD/Shi-scid IL-2γnul (NOG) mice, which were transplantated with 2 × 105 My-La cells and died owing to tumor cell invasion and metastasis to multiple visceral organs/tissues during day 29–35 after the transplantation [10, 11, 18]. We used this *in vivo* mouse model to further determine whether miR-150 inhibits tumor metastasis. We have shown that CTCL cells could produce IL-22 [10], and significant increases in serum IL-22 levels in patients with CTCL, especially in advanced cases [23]. Because serum IL-22 levels positively correlated with disease activity in CTCL, IL-22 concentration in peripheral blood of the transplanted NOG mice could be an index of invasion and metastasis. Therefore, we examined the IL-22 production of the CTCL cell- transplantated mice and discovered that it started increasing after day 14 from the transplantation of the CTCL cells into the NOG mice. Figure 6A shows the enzyme-linked immunosorbent assay (ELISA) of IL-22 in transduced My-La cells. This data demonstrated that when the serum IL-22 concentration increased to approximately 2,000–2,500 pg/mL, the NOG mice would die because of invasion and metastasis to multiple organs. Based on this data, we decided to inject the miRNA or small-interfering RNA (siRNA) after day 14 from CTCL cell transplantation. We administered miR-150 or siCCR6 (30 μM each) to the CTCL mice via the tail vein every 5 days. These agents were conjugated with atelocollagen prior to administration as described previously [11, 32]. The administration significantly prolonged survival when compared with that in the control; however, we found that miR-150 administration prolonged the survival of CTCL mice more significantly than siCCR6 did (Figure 6B). We previously reported that xenografted mice transplanted with miR-150-transduced CTCL cells inhibited the invasion and metastasis and prolonged their survival [10]. Thus, administration of miR-150 may have prolonged mouse survival through a metastasis inhibition mechanism.

## DISCUSSION

We found that miR-150 was downregulated only in advanced MF cases, and its expression was restored by pan-HDACI treatment, suggesting that this effect might be mediated by epigenetic silencing mechanisms of miR-150 during the progression of MF. Moreover, when we consider our previous findings that downregulation of miR-16 was occurred even in early stage of MF that was also restored by vorinostat [18], dysregulated miRNAs by HDACs should be crucially involved in the pathogenesis of MF throughout the early and advanced stages. This downregulation is likely due to HDAC activation throughout the progression of MF. Recently, HDAC activation was shown to also occur in various lymphoma subtypes [33, 34]. Therefore, these results bring us another question which mechanism can induce HDACs’ activation. Recent reports have possibly addressed the potential underlying mechanism by suggesting that aberrant DNA mutation of histone acetyltransferases (HAT) encodes genes that frequently occur in various lymphoma subtypes [35].

For instance, in B-cell lymphomas approximately 39 and 41% of the diffuse large B-cell and follicular lymphoma cases, respectively display genomic deletions, somatic mutations, or both that remove or inactivate the HAT coding domain of the cyclic adenosine monophosphate (cAMP) response element-binding protein (CREB) binding protein (CREBBP) and adenovirus early region 1A (E1A) binding protein P300 (EP300). The HAT and HDAC expression balance is exact and accurately regulated in normal lymphocytes. However, in malignant cells, we speculate that HAT alterations could result in the relatively increased activity of the HDACs, leading to the downregulation of tumor suppressor genes or tumor suppressive miRNAs. Considering that the mechanisms of HDAC inhibition in patients with CTCL is poorly understood, the HAT/HDAC genetic alterations were of specific interest. Furthermore, three groups recently reported on the genomic landscape of genetic mutations in CTCL using whole-exome sequencing analysis, which might address this problem of lack of understanding [36–38].

Interestingly, da Silva Almeida and colleagues [37] showed that structural mutations in the HDACs were not detected while the loss-of-function mutations in the CREBBP were detected only in 4.8% of the samples. Therefore, we cannot conclude that HAT inactive-mutation is a general major cause of HDAC activation in CTCL. Another possibility is that there may be a critical novel cascade that regulates HDACs downstream of these oncogenic products, because there are several active mutations of the oncogenic transcription factors in CTCL [39]. Indeed, it has been demonstrated that the Myc transcription factor, which is frequently dysregulated in almost all advanced cancers, has the potential to recruit HDAC3 in mantle cell lymphoma [40]. Because Myc is also overexpressed in CTCL cell lines and advanced CTCL cases (Supplementary Figure S1), the activation of the Myc-HDAC cascade may also be a possible mechanism of the aberrant HDAC activations. The detailed mechanism underlying HDAC activation in CTCL is still unclear. Thus, the elucidation of the exact oncogenic cascade that activates the HDACs is warranted for future study.

Although some HDACIs are currently being developed or investigated for use in non-Hodgkin's lymphomas including CTCL [19–22], there is a lack of evidence supporting the efficacy of HDACIs, and a specific HDACI that may be effective against advanced CTCL has not been identified. However, we found that pan-HDACIs such as vorinostat and panobinostat, which inhibited Class I and II HDACs, effectively inhibited metastasis. In contrast, romidepsin, which specifically inhibits HDAC1 and HDAC2, did not inhibit CTCL cell migration. These results suggest that inhibition of other Class I HDACs such as HDAC3, HDAC8, or both is necessary to inhibit migration. The target genes of the HDAC may not only share a common gene, but also have distinct target genes. Moreover, each HDAC may activate tumor suppressor genes and oncogenes. This suggests that a precise balance of the activation level of each HDAC is required to effectively suppress CTCL cells. Indeed, Geoffrey et al. [41] showed that the combined depletion of HDAC1 and HDAC2 reduced the level of Eμ-Myc cells to a greater extent than single isoform depletion in Eμ-Myc lymphoma, suggesting the importance of concomitant inhibition of HDACs. Additional detailed studies of the specific combinations of HDACs that would most effectively inhibit migration are also required.

In this study, we reported that the HDACIs not only restored the miRNAs associated with migration but also other miRNAs that might possess tumor suppressive functions. The HDACIs upregulated well-known tumor suppressive miRNAs including miR-16, miR-22, miR-192-194-215 cluster, and miR-320 family. For instance, we previously demonstrated that miRNA induced apoptosis or senescence in CTCL cells [18]. Furthermore, miR-22 was also demonstrated as a tumor suppressive factor in CTCL pathogenesis [42]. In other tumors, it has been demonstrated that the enhanced expression of the miR-192-194-215 cluster, is tumor suppressive in combination with p53 in multiple myeloma [43]. The downregulation of the miR-320 family in the microenvironment represents an oncogenic phenotype of breast cancer [44]. In addition to metastasis inhibition, the restoration of the tumor suppressive miRNAs by HDACIs could contribute to inhibiting the growth and survival of CTCL cells.

Our data suggest that miR-150 is a candidate with prognostic value. Previously, signal transducer and activator of transcription 3 (STAT3) was also proposed a prognostic marker for predicting the responsiveness of patients with CTCL to HDACIs [45]. Our study further suggests that miR-150 could not only be a powerful biomarker for molecular diagnosis and a predictor of metastasis, but it could also be a novel therapeutic agent for use in advanced CTCL. The quantitative evaluation of miR-150 might be useful for determining the appropriate time of therapeutic intervention in the transformation from the early to the advanced MF state. Vorinostat has been shown to contribute significantly to a superior survival than chemotherapy only in the advanced-stage of MF [46], suggesting that HDACI may be useful for cases with downregulation of miR-150. Therefore, the miRNA expression analysis using qRT-PCR may be advantageous for diagnosis because miRNA can be obtained from paraffin-embedded tissue, and may not be affected by analytical or other conditions. However, because the microdissection technology for CD4^+^ cells is not well established in Research Institutes or Hospitals, the establishment of diagnostic and validation criteria with more cohorts would be required in further studies.

In conclusion, we strongly suggest that miRNAs dysregulation is crucially involved in CTCL pathogenesis and could be induced by the activation of HDAC during the progression of CTCL.

## MATERIALS AND METHODS

### Primary lymphoma samples

We collected RNA samples from paraffin embedded skin biopsy specimens from patients with early (patch and/or plaque phase, *n* = 26) and advanced (tumor phase, *n* = 14) CTCL. Detailed information was described in previous published paper [18]. The ISCL-EORTC (International Society for Cutaneous Lymphomas-European Organization for Research and Treatment of Cancer) diagnostic criteria for cutaneous lymphomas were used for clinical staging of CTCL [1–3]. Written informed consent was obtained from all patients prior to collection of specimens, in keeping with all institutional policies and according to the Declaration of Helsinki. Samples were collected under a protocol approved by the Institutional Review Boards of Akita University (no.1313) and University of Tokyo (no.10746). Patient information (age, sex, histology, stage, therapy and others) is described at Supplementary Table S3.

### Micro dissection

Regions that were stained by CD4 (the region should be contain CD4^+^ cells with high concentration) were microscopically dissected out and examined for miRNA expression. Micro-dissection was conducted against paraffin embedded tissue samples by use of LMD6500 (Leica, Japan).

### Cell lines

HH, HUT78, and MJ cell lines were purchased from the American Type Tissue Collection (ATCC). My-La was from the European Collection of Cell Cultures (ECACC). My-La, MJ, HH, and HUT78 were cultured in Arteimis-1 medium (with or without 2% inactivated human serum), which is a chemically defined, serum-free medium purchased from NihonTechno Service Co. Ltd. (Ibaraki, Japan). It contains recombinant human Insulin (5.0 μg/L), recombinant human IL-2 (250 IU/mL), human serum albumin (2 g/L) and no other cytokines nor growth factors are contained.

### Cell migration assay

*In vitro* cell migration assay was carried out by use of CytoSelect 96-Well Cell Migration Assay kit (5 μm, Fluorometric Format) (Cell Biolabs, Inc. SanDiego, CA, USA) according to the manufacturer's protocol.

### miRNA microarray analysis

We analyzed miRNA expression using an Agilent miRNA microarray (Rel.19.0, G4872A), (Agilent, Santa Clara, CA). The array scanner was an Agilent G2600A SureScan Microarray Scanner System, or Agilent Protocol Ver 2.4 (for miRNA expression). Data were analyzed by GeneSpring (Agilent). Data were updated at GSE81190.

### Transient miRNA transfection

12 miRNAs (mirVanaTM miRNA mimics) and negative control miRNA were purchased from Life Technologies (Life Technology Japan LTd). The transfection of miRNA was used by the Nucleofector II and the Cell Line Nucleofector Kit V (VCA-1003) (Amaxa, Koeln, Germany) according to the manufacturer's protocol. Transduction efficacy of miR-150 against CTCL cell lines was described in our previous report [10].

### *In vivo* administration of mi/siRNA to xenografted mice

Mature miR-150 (AteloSiLence^®^) was purchased from KOKEN CO., LTD (Tokyo, Japan). My-La (2 × 10^5^ cells each) was subcutaneously injected into the right or left side of the body of 6- to 8-week-old female NOG mice (Central Institute for Experimental Animals, Kawasaki, Japan) [10, 11, 18]. 30 μM of siCCR6 or miR-150 plus atelocollagen (200 μl) (AteloGene^®^ Local Use “Quick Gelation” kit. KOKEN CO., LTD, Tokyo, Japan, ref 32) was injected into tail vein of NOG mice after day 14 from CTCL cell transplantation (2 × 10^5^ My-La cells/per body) by every 5 days.

### Chemicals

Vorinostat, panobinostat, romidepsin were purchased from R&D systems, RG108, and nutlin-3a was from Cosmo Bio Co., LTD (Japan).

Detailed Methods of reverse transcription-polymerase chain reaction (qRT-PCR), Northern blot analysis, Western blot analysis, Immunohistochemical analysis and others are described in Supplementary Materials and Methods.

## SUPPLEMENTARY MATERIALS FIGURES AND TABLES







## References

[1] Willemze R, Jaffe ES, Burg G, Cerroni L, Berti E, Swerdlow SH, Ralfkiaer E, Chimenti S, Diaz-Perez JL, Duncan LM, Grange F, Harris NL, Kempf W (2005). WHO-EORTC classification for cutaneous lymphomas. Blood.

[2] Olsen Elise, Vonderheid Eric, Pimpinelli Nicola, Willemze Rein, Kim Youn, Knobler Robert, Zackheim Herschel, Duvic Madeleine, Estrach Teresa, Lamberg Stanford, Wood Gary, Dummer Reinhard, Ranki Annamari (2007). Revisions to the staging and classification of mycosis fungoides and Sezary syndrome: a proposal of the International Society for Cutaneous Lymphomas (ISCL) and the cutaneous lymphoma task force of the European Organization of Research and Treatment of Cancer (EORTC). Blood.

[3] Ralfkiaer E, Cerroni L, Sander CA, Smoller BR, Willemze R, Swerdlow SH, Campo E, Harris NL, Jaffe ES, Pileri SA, Stein H, Thiele J, Vardiman JW (2008). Mycosis fungoides.. WHO Classification of Tumors of Haematopoietic and Lymphoid Tissues.

[4] Lewis BP, Burge CB, Bartel DP (2005). Conserved seed pairing, often flanked by adenosines, indicates that thousands of human genes are microRNA targets. Cell.

[5] Bartel DP (2009). MicroRNAs: target recognition and regulatory functions. Cell.

[6] Croce CM (2009). Causes and consequences of microRNA dysregulation in cancer. Nat Rev Genet.

[7] Tagawa H, Ikeda S, Sawada K (2013). Role of microRNA in the pathogenesis in malignant lymphoma. Cancer Sci.

[8] Ikeda S, Tagawa H (2014). Dysregulation of microRNAs and their association in the pathogenesis of T-cell lymphoma/leukemias. Int J Hematol.

[9] Watanabe A, Tagawa H, Yamashita J, Teshima K, Nara M, Iwamoto K, Kume M, Kameoka Y, Takahashi N, Nakagawa T, Shimizu N, Sawada K (2011). The role of microRNA-150 as a tumor suppressor in malignant lymphoma. Leukemia.

[10] Ito M, Teshima K, Ikeda S, Kitadate A, Watanabe A, Nara M, Yamashita J, Ohshima K, Sawada K, Tagawa H (2014). MicroRNA-150 inhibits tumor invasion and metastasis by targeting the chemokine receptor CCR6, in advanced cutaneous T-cell lymphoma. Blood.

[11] Ikeda S, Kitadate A, Ito M, Abe F, Nara M, Watanabe A, Takahashi N, Miyagaki T, Sugaya M, Tagawa H (2016). Disruption of CCL20-CCR6 interaction inhibits metastasis of advanced cutaneous T-cell lymphoma. Oncotarget.

[12] Lane AA, Chabner BA (2009). Histone deacetylase inhibitors in cancer therapy. J Clin Oncol.

[13] Duvic M, Talpur R, Ni X, Zhang C, Hazarika P, Kelly C, Chiao JH, Reilly JF, Ricker JL, Richon VM, Frankel SR (2007). Phase 2 trial of oral vorinostat (suberoylanilide hydroxamic acid, SAHA) for refractory cutaneous T-cell lymphoma (CTCL). Blood.

[14] Olsen EA, Kim YH, Kuzel TM, Pacheco TR, Foss FM, Parker S, Frankel SR, Chen C, Ricker JL, Arduino JM, Duvic M (2007). Phase IIb multicenter trial of vorinostat in patients with persistent, progressive, or treatment refractory cutaneous T-cell lymphoma. J Clin Oncol.

[15] Richon VM, Sandhoff TW, Rifkind RA, Marks PA (2000). Histone deacetylase inhibitor selectively induces p21WAF1 expression and gene-associated histone acetylation. Proc Natl Acad Sci USA.

[16] Gui CY, Ngo L, Xu WS, Richon VM, Marks PA (2004). Histone deacetylase (HDAC) inhibitor activation of p21WAF1 involves changes in promoter-associated proteins, including HDAC1. Proc Natl Acad Sci USA.

[17] Marks PA, Breslow R (2007). Dimethyl sulfoxide to vorinostat: development of this histone deacetylase inhibitor as an anticancer drug. Nat Biotech.

[18] Kitadate A, Ikeda S, Teshima K, Ito M, Toyota I, Hasunuma N, Takahashi N, Miyagaki T, Sugaya M, Tagawa H (2016). MicroRNA-16 mediates the regulation of a senescence-apoptosis switch in cutaneous T-cell and other non-Hodgkin lymphomas. Oncogene.

[19] Richardson PG, Hungria VT, Yoon SS, Beksac M, Dimopoulos MA, Elghandour A, Jedrzejczak WW, Guenther A, Nakorn TN, Siritanaratkul N, Schlossman RL, Hou J, Moreau P (2016). Panobinostat plus bortezomib and dexamethasone in relapsed/relapsed and refractory myeloma: outcomes by prior treatment. Blood.

[20] Tan D, Phipps C, Hwang WY, Tan SY, Yeap CH, Chan YH, Tay K, Lim ST, Lee YS, Kumar SG, Ng SC, Fadilah S, Kim WS (2015). SGH651 investigators. Panobinostat in combination with bortezomib in patients with relapsed or refractory peripheral T-cell lymphoma: an open-label, multicentre phase 2 trial. Lancet Haematol.

[21] Prince HM, Dickinson M (2012). Romidepsin for cutaneous T-cell lymphoma. Clin Cancer Res.

[22] Coiffier B, Pro B, Prince HM, Foss F, Sokol L, Greenwood M, Caballero D, Borchmann P, Morschhauser F, Wilhelm M, Pinter-Brown L, Padmanabhan S, Shustov A (2012). Results from a pivotal, open-label, phase II study of romidepsin in relapsed or refractory peripheral T-cell lymphoma after prior systemic therapy. J Clin Oncol.

[23] Miyagaki T, Sugaya M, Suga H, Kamata M, Ohmatsu H, Fujita H, Asano Y, Tada Y, Kadono T, Sato S (2011). IL-22, but not IL-17, dominant environment in cutaneous T-cell lymphoma. Clin Cancer Res.

[24] Rubie C, Frick VO, Ghadjar P, Wagner M, Grimm H, Vicinus B, Justinger C, Graeber S, Schilling MK (2010). CCL20/CCR6 expression profile in pancreatic cancer. J Transl Med.

[25] Kirshberg S, Izhar U, Amir G, Demma J, Vernea F, Beider K, Shlomai Z, Wald H, Zamir G, Shapira OM, Peled A, Wald O (2011). Involvement of CCR6/CCL20/IL-17 axis in NSCLC disease progression. PLoS One.

[26] Wang L, Qin H, Li L, Zhang Y, Tu Y, Feng F, Ji P, Zhang J, Li G, Zhao Z, Gao G (2012). Overexpression of CCL20 and its receptor CCR6 predicts poor clinical prognosis in human gliomas. Med Oncol.

[27] Frick VO, Rubie C, Kölsch K, Wagner M, Ghadjar P, Graeber S, Glanemann M (2013). CCR6/CCL20 chemokine expression profile in distinct colorectal malignancies. Scand J Immunol.

[28] Tovar C, Rosinski J, Filipovic Z, Higgins B, Kolinsky K, Hilton H, Zhao X, Vu BT, Qing W, Packman K, Myklebost O, Heimbrook DC, Vassilev LT (2006). Small-molecule MDM2 antagonists reveal aberrant p53 signaling in cancer: implications for therapy. Proc Natl Acad Sci USA.

[29] Manfé V, Biskup E, Johansen P, Kamstrup MR, Krejsgaard TF, Morling N, Wulf HC, Gniadecki R (2012). MDM2 Inhibitor Nutlin-3a induces apoptosis and senescence in cutaneous T-cell lymphoma: Role of p53. J Invest Dermatol.

[30] Litvinov IV, Cordeiro B, Huang Y, Zargham H, Pehr K, Doré MA, Gilbert M, Zhou Y, Kupper TS, Sasseville D (2014). Ectopic expression of cancer-testis antigens in cutaneous T-cell lymphoma patients. Clin Cancer Res.

[31] Hoareau-Aveilla C, Valentin T, Daugrois C, Quelen C, Mitou G, Quentin S, Jia J, Spicuglia S, Ferrier P, Ceccon M, Giuriato S, Gambacorti-Passerini C, Brousset P (2015). Reversal of microRNA-150 silencing disadvantages crizotinib-resistant NPM-ALK(+) cell growth. J Clin Invest.

[32] Ochiya T, Takahama Y, Nagahara S, Sumita Y, Hisada A, Itoh H, Nagai Y, Terada M (1999). New delivery system for plasmid DNA in vivo using atelocollagen as a carrier material: the Minipellet. Nat Med.

[33] Zain J, O'Connor OA (2010). Targeting histone deacetyalses in the treatment of B- and T-cell malignancies. Invest New Drugs.

[34] Zhang X, Chen X, Lin J, Lwin T, Wright G, Moscinski LC, Dalton WS, Seto E, Wright K, Sotomayor E, Tao J (2012). Myc represses miR-15a/miR-16-1 expression through recruitment of HDAC3 in mantle cell and other non-Hodgkin B-cell lymphomas. Oncogene.

[35] Pasqualucci L, Dominguez-Sola D, Chiarenza A, Fabbri G, Grunn A, Trifonov V, Kasper LH, Lerach S, Tang H, Ma J, Rossi D, Chadburn A, Murty VV (2011). Inactivating mutations of acetyltransferase genes in B-cell lymphoma. Nature.

[36] Choi J, Goh G, Walradt T, Hong BS, Bunick CG, Chen K, Bjornson RD, Maman Y, Wang T, Tordoff J, Carlson K, Overton JD, Liu KJ (2015). Genomic landscape of cutaneous T cell lymphoma. Nat Genet.

[37] da Silva Almeida AC, Abate F, Khiabanian H, Martinez-Escala E, Guitart J, Tensen CP, Vermeer MH, Rabadan R, Ferrando A, Palomero T (2015). The mutational landscape of cutaneous T cell lymphoma and Sézary syndrome. Nat Genet.

[38] Ungewickell A, Bhaduri A, Rios E, Reuter J, Lee CS, Mah A, Zehnder A, Ohgami R, Kulkarni S, Armstrong R, Weng WK, Gratzinger D, Tavallaee M (2015). Genomic analysis of mycosis fungoides and Sezary syndrome identifies recurrent alterations in TNFR2. Nat Genet.

[39] Woollard WJ, Pullabhatla V, Lorenc A, Patel VM, Butler RM, Bayega A, Begum N, Bakr F, Dedhia K, Fisher J, Aguilar-Duran S, Flanagan C, Ghasemi AA (2016). Candidate driver genes in Sézary syndrome: frequent perturbations of genes involved in genome maintenance and DNA repair. Blood.

[40] Zhang X, Zhao X, Fiskus W, Lin J, Lwin T, Rao R (2012). Coordinated silencing of MYC-mediated miR-29 by HDAC3 and EZH2 as a therapeutic target of histone modification in aggressive B-Cell lymphomas. Cancer Cell.

[41] Matthews GM, Mehdipour P, Cluse LA, Falkenberg KJ, Wang E, Roth M, Santoro F, Vidacs E, Stanley K, House CM, Rusche JR, Vakoc CR, Zuber J (2015). Functional-genetic dissection of HDAC dependencies in mouse lymphoid and myeloid malignancies. Blood.

[42] Sibbesen NA, Kopp KL, Litvinov IV, Jønson L, Willerslev-Olsen A, Fredholm S, Petersen DL, Nastasi C, Krejsgaard T, Lindahl LM, Gniadecki R, Mongan NP, Sasseville D (2015). Jak3, STAT3, and STAT5 inhibit expression of miR-22, a novel tumor suppressor microRNA, in cutaneous T-Cell lymphoma. Oncotarget.

[43] Pichiorri F, Suh SS, Rocci A, L De Luca, Taccioli C, Santhanam R, Zhou W, Benson DM, Hofmainster C, Alder H, Garofalo M, G Di Leva, Volinia S (2010). Downregulation of p53-inducible microRNAs 192, 194, and 215 impairs the p53/MDM2 autoregulatory loop in multiple myeloma development. Cancer Cell.

[44] Bronisz A, Godlewski J, Wallace JA, Merchant AS, Nowicki MO, Mathsyaraja H, Srinivasan R, Trimboli AJ, Martin CK, Li F, Yu L, Fernandez SA, Pécot T (2011). Reprogramming of the tumour microenvironment by stromal PTEN-regulated miR-320. Nat Cell Biol.

[45] Fantin VR, Loboda A, Paweletz CP, Hendrickson RC, Pierce JW, Roth JA (2008). Constitutive activation of signal transducers and activators of transcription predicts vorinostat resistance in cutaneous T-cell lymphoma. Cancer Res.

[46] Hughes CF, Khot A, McCormack C, Lade S, Westerman DA, Twigger R (2015). Lack of durable disease control with chemotherapy for mycosis fungoides and Sézary syndrome: a comparative study of systemic therapy. Blood.

