# Supplementary material for: Histone deacetylase inhibitors inhibit metastasis by restoring a tumor suppressive microRNA-150 in advanced cutaneous T-cell lymphoma
Source: Oncotarget. 2016 Dec 7;8(5):7572–85. doi: 10.18632/oncotarget.13810 (PMC5352344; doi:10.18632/oncotarget.13810)
Supplement: Supplementary file 1 [file oncotarget-08-7572-s001.pdf]

Histone deacetylase inhibitors inhibit metastasis by restoring a tumor suppressive microRNA-150 in advanced cutaneous T-cell lymphoma

Supplementary Materials

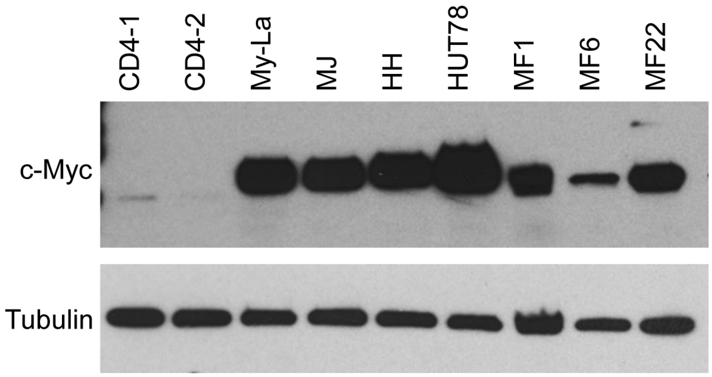

Supplementary Figure S1: Western blot analysis of Myc in CTCL cell lines and three cases of primary and advanced CTCL, which were obtained from lymph nodes of patients with CTCL tumors (MF1, 6, and 22). Tubulin is a control.

Supplementary Table S1: List of 161 miRNAs that were commonly upregulated by both vorinostat and panobinostat in My-La, HH and HUT78. See Supplementary_Table_S1

Supplementary Table S2: Northern blot analysis for HDACis induced miRNA that possesses seed sequence of CCR6. See Supplementary_Table_S2

Supplementary Table S3: Information of patients with MF

Case	Age (at first diagnosis)	Sex	Histology at first diagnosis	Stage at first diagnosis	Treatment and Therapy	Duration from early to tumor phase (months)
MF1	45	M	patch	I A	nbUVB[#1] + etretinate, EBRT[#2] + surgery	50
MF2	57	M	patch	I A	no therapy	NA[#3]
MF3	51	M	patch	I A	no therapy	NA
MF4	48	F	patch	I A	nbUVB	NA
MF5	71	M	patch	I A	PSL[#4]	NA
MF6	67	M	plaque	I B	nbUVB + etretinate, EBRT	54
MF7	71	M	patch and plaque	I B	nbUVB + (bexarotene→etretinate), EBRT	30
MF8	55	M	plaque	I B	nbUVB + etretinate, EBRT	NA
MF9	59	M	patch and plaque	I B	PUVA[#5]	NA
MF10	38	M	patch	I B	nbUVB	NA
MF11	26	F	patch and plaque	I B	nbUVB	NA
MF12	70	M	patch and plaque	I B	nbUVB	NA
MF13	64	M	patch and plaque	I B	nbUVB	NA
MF14	61	M	plaque	I B	(nbUVB→PUVA) + etretinate, EBRT→CHOP[#6]	NA
MF15	61	M	plaque	I B	PSL	NA
MF16	76	F	patch	I B	PSL	NA
MF17	62	M	patch and plaque	I B	PSL	NA
MF18	27	F	patch	I B	PSL	NA
MF19	78	F	patch	I B	PSL	NA
MF20	64	F	patch and plaque	II A	EBRT, PSL, IFN-gamma, etoposide→ nbUVB + (etretinate→etoposide→CPA[#7])	NA
MF21	62	M	plaque	II A	nbUVB + (etretinate→MTX[#8]→etoposide)	NA
MF22	40	M	patch	II A	IFN-gamma, PSL, PUVA, radiation	170
MF23	73	M	plaque and tumor	II B	PUVA	0
MF24	71	M	plaque and tumor	II B	nbUVB+PSL+ (etretinate→IFN-gamma→etoposide)	0
MF25	24	M	plaque and tumor	II B	CHOP+allo-BMT[#9]	0
MF26	63	F	tumor	II B	nbUVB+ (MTX→etoposide) + PSL→gemcitabine + PSL→mogamulizumab+PSL	NA
MF27	20	M	tumor	II B	IFN-gamma, PUVA, PSL, nbUVB, radiation vorinostat	NA
MF28	62	M	plaque and tumor	III	nbUVB + etretinate	0
MF29	75	M	tumor	IV	nbUVB + etretinate + PSL	NA
MF30	64	M	tumor	IV	nbUVB→CHOP	NA
MF31	41	M	tumor	IV	PSL→nbUVB→ vorinostat	NA
MF32	24	M	tumor	IV	PSL→nbUVB→ vorinostat	NA

#1, nbUVB: narrow band UV beta wave therapy
#2, EBI: total skin electron beam radiation therapy
#3, NA: not applicable
#4, PSL: prednisolone
#5, PUVA: psoralen ultraviolet A treatment
#6, CHOP: CPA, hydroxidoxiorubicin, vincristin, PSL therapy
#7, cyclophosphamide; CPA
#8, BMT: bone marrow transplantation
#9, MTX: methotrexate
