# Supplementary material for: Histone deacetylase inhibitors inhibit metastasis by restoring a tumor suppressive microRNA-150 in advanced cutaneous T-cell lymphoma
Source: Oncotarget. 2016 Dec 7;8(5):7572–85. doi: 10.18632/oncotarget.13810 (PMC5352344; doi:10.18632/oncotarget.13810)
Supplement: Supplementary file 2 [file oncotarget-08-7572-s002.docx]

| Table S1. List of 161 miRNAs that were commonly upregulated by both vorinostat and panobinostat in My-La, HH and HUT78. | | | | | | | | | | | | |  |  |  |  |  |  |
| --- | --- | --- | --- | --- | --- | --- | --- | --- | --- | --- | --- | --- | --- | --- | --- | --- | --- | --- |
|  |  |  |  | Fold change | | | | | | | |  |  |  |  |  |  |  |
|  | Function | |  | SAHA | | |  | panobinostat | | |  |  |  |  |  |  |  |  |
| microRNA | TSG | OG |  | My-La | HH | HUT78 |  | MyLa | HH | HUT78 | mean | function | target | cancer type | ref1 | ref2 | ref3 |  |
| let-7b-5p | ○ | - |  | 1.8052338 | 2.615865 | 6.828541 |  | 1.692826 | 3.0020113 | 5.0991135 | 3.5072651 | cell cycle arrest | CDK6 CCND1 HMGA2 | lung cancer (Johnson et al) melanoma (Schltz et al) rhabdoid tumor (Zhang et al) | Johnson CD, et al. The let-7 microRNA represses cell proliferation pathways in human cells. *Cancer Res* 2007;67:7713-7722. | Schultz J, et al. MicroRNA let-7b targets important cell cycle molecules in malignant melanoma cells and interferes with anchorage-independent growth. *Cell Res* 2008;18:549-557. | Zhang K, et al. Frequent overexpression of HMGA2 in human atypical teratoid/rhabdoid tumor and its correlation with let-7a3/let-7b miRNA. *Clin Cancer Res* 2014;20:1179-1189. |  |
| miR-16-5p | ○ | - |  | 1.7454602 | 2.1309469 | 2.528478 |  | 1.6692126 | 1.9244314 | 1.7614516 | 1.959996783 | cell cycle arrest apoptosis induction | CCND1 CDK6 BMI1 | prostate cancer (Bonci et al) mantle cell lymphoma (Teshima et al) cutaneous T-cell lymphoma (Kitadate et al) | Bonci D, et al. The miR-15a-miR-16-1 cluster controls prostate cancer by targeting multiple oncogenic activities. *Nat Med* 2008;14:1271-1277. | Teshima K, et al. Dysregulation of BMI1 and microRNA-16 collaborate to enhance an anti-apoptotic potential in the side population of refractory mantle cell lymphoma. *Oncogene* 2014;33:2191-2203. | Kitadate A, et al. MicroRNA-16 mediates the regulation of a senescence-apoptosis switch in Cutaneosu T-cell and other non Hodgkin lymphomas. *Oncogene* 2015;Epub ahead of print. |  |
| miR-22-3p | ○ | - |  | 6.641563 | 13.505392 | 14.173419 |  | 6.1398296 | 10.633375 | 13.578965 | 10.77875727 | cell growth inhibition | FLT3 | cutanoeus T-cell lymphoma (Sibbese et al) acute myeloid leukemia (Jiang et al) | Sibbesen NA, et al. Jak3, STAT3, and STAT5 inhibit expression of miR-22, a novel tumor suppressor microRNA, in cutaneous T-Cell lymphoma. *Oncotarget* 2015;6:20555-20569. | Jiang X, et al. miR-22 has a potent anti-tumour role with therapeutic potential in acute myeloid leukaemia. *Nat commun* 2016;7:11452. |  |  |
| miR-23a-3p | ○ | ○ |  | 2.5950844 | 4.1588902 | 2.5580177 |  | 2.1832995 | 3.3227758 | 1.6698692 | 2.747989467 | metastasis and invasion metastasis inhibition | MTSS1 PAK6 | colorectal cancer (Jahid et al) prostate cancer (Cai et al) | Jahid S, et al. miR-23a promotes the transition from indolent to invasive colorectal cancer. *Cancer Discov* 2012;2:540-553. | Cai S, et al. Downregulation of microRNA-23a suppresses prostate cancer metastasis by targeting the PAK6-LIMK1 signaling pathway. *Oncotarget* 2015;6:3904-3917. | - |  |
| miR-26a-5p | ○ | - |  | 5.219224 | 3.6554706 | 2.7063017 |  | 4.974843 | 2.6209035 | 1.7846025 | 3.49355755 | cell cycle arrest metastasis inhibition | EZH2 PTGS2 | burkitt lymphoma (Sander et al) | Sander S, et al. MYC stimulates EZH2 expression by repression of its negative regulator miR-26a. *Blood* 2008;112:4202-4212. | Shao Y, et al. MiR-26a and miR-144 inhibit proliferation and metastasis of esophageal squamous cell cancer by inhibiting cyclooxygenase-2. *Oncotarget* 2016:Epub ahead of print. | - |  |
| miR-26b-5p | ○ | - |  | 1.8465977 | 2.7520924 | 2.7915626 |  | 1.7427019 | 2.0697372 | 1.7829329 | 2.164270783 | metastasis inhiibition | KPNA2 | gastric cancer (Tsai et al) | Tsai MM, et al. MicroRNA-26b inhibits tumor metastasis by targeting the KPNA2/c-jun pathway in human gastric cancer. *Oncotarget* 2016;Epub ahead of print. | - | - |  |
| miR-27b-3p | ○ | ○ |  | 2.31305 | 2.2327063 | 2.0348563 |  | 2.143743 | 1.7636875 | 1.5954763 | 2.0139199 | cell growth inhibition | PPARγ NISCH | neuroblastoma (Lee et al) breast cancer (Jin et al) | Lee JJ, et al. MiR-27b targets PPARγ to inhibit growth, tumor progression and the inflammatory response in neuroblastoma cells. *Oncogene* 2012;31:3818-3825. | Jin L, et al. Prooncogenic factors miR-23b and miR-27b are regulated by Her2/Neu, EGF, and TNF-α in breast cancer. *Cancer Res* 2013;73:2884-2896. | - |  |
| miR-30c-5p | ○ | - |  | 2.177809 | 2.138255 | 2.1934206 |  | 1.8814967 | 1.8005117 | 1.5522791 | 1.95729535 | cell growth inhibition | KRAS NOTCH1 | breast cancer (Tanic et al) acute myeloid leukemia (Katzeke et al) | Tanic M, et al. Deregulated miRNAs in hereditary breast cancer revealed a role for miR-30c in regulating KRAS oncogene. *PLoS One* 2012;7:e38847. | Katzerke C, et al. Transcription factor C/EBPα-induced microRNA-30c inactivates Notch1 during granulopoiesis and is downregulated in acute myeloid leukemia. *Blood* 2013;122:2433-2442. | - |  |
| miR-30d-5p | ○ | - |  | 2.2735913 | 2.3838174 | 2.3328586 |  | 1.9855812 | 2.1964462 | 1.6786495 | 2.141824033 | apoptosis induction | GRP78 | prostate cancer (Su et al) lung cancer (Chen et al) | Su SF, et al. miR-30d, miR-181a and miR-199a-5p cooperatively suppress the endoplasmic reticulum chaperone and signaling regulator GRP78 in cancer. *Oncogene* 2013;32:4694-4701. | Chen D, et al. MicroRNA-30d-5p inhibits tumour cell proliferation and motility by directly targeting CCNE2 in non-small cell lung cancer. *Cancer Lett* 2015;362:208-217. | - |  |
| miR-30e-5p | ○ | - |  | 2.159063 | 2.054538 | 2.425641 |  | 2.0246575 | 1.74293 | 1.6691706 | 2.012666683 | apoptosis induction | ABL | chronic myeloid leukemia (Hershkovitz et al) | Hershkovitz-Rokah O, et al. MiR-30e induces apoptosis and sensitizes K562 cells to imatinib treatment via regulation of the BCR-ABL protein. *Cancer Lett* 2015;356:597-605. | - | - |  |
| miR-32-3p | - | - |  | 31.731379 | 61.538303 | 277.15308 |  | 9.999999 | 49.391804 | 220.03424 | 108.3081342 | - | - | - | - | - | - |  |
| miR-96-5p | ○ | - |  | 165.91768 | 9.382522 | 6.5175877 |  | 134.32791 | 7.960973 | 6.992182 | 55.18314245 | apoptosis induction | KRAS HERG1 | pancreatic cancer (Yu et al, Feng et al) | Yu S, et al. miRNA-96 suppresses KRAS and functions as a tumor suppressor gene in pancreatic cancer. *Cancer Res* 2010;70:6015-6025. | Feng J, et al. HERG1 functions as an oncogene in pancreatic cancer and is downregulated by miR-96. *Oncotarget* 2014;5:5832-5844. | - |  |
| miR-107 | ○ | ○ |  | 1.6979146 | 2.7856224 | 2.1689851 |  | 1.6231543 | 2.285103 | 1.5155131 | 2.012715417 | angiogenesis inhibition metastasis cell growth | HIF-1β DAPK CPEB3 | colon cancer (Yamakuchi et al) colorectal cancer (Chen et al) hepatocellular carcinoma (Zou et al) | Yamakuchi M, et al. P53-induced microRNA-107 inhibits HIF-1 and tumor angiogenesis. *Proc Natl Acad Sci U S A*. 2010;107:6334-6339. | Chen HY, et al. miR-103/107 promote metastasis of colorectal cancer by targeting the metastasis suppressors DAPK and KLF4. *Cancer Res* 2012;72:3631-3641. | Zou CD, et al. MicroRNA-107: a novel promoter of tumor progression that targets the CPEB3/EGFR axis in human hepatocellular carcinoma. *Oncotarget* 2016;7:266-278. |  |
| miR-132-3p | ○ | - |  | 2.76179 | 50.675903 | 2.948724 |  | 2.5057116 | 25.689203 | 2.1880436 | 14.46156253 | metastasis inhibition | TALIN2 ZEB2 | prostate cancer (Formosa et al) lung cancer ( You et al) | Formosa A, et al. DNA methylation silences miR-132 in prostate cancer. *Oncogene* 2013;32:127-134. | You J, et al. MiR-132 suppresses the migration and invasion of lung cancer cells via targeting the EMT regulator ZEB2. *PLoS One* 2014;9:e91827. | - |  |
| miR-134 | ○ | ○ |  | 89.54388 | 143.23952 | 80.78229 |  | 72.35558 | 155.6731 | 55.723698 | 99.55301133 | metastasis cell growth inhibtion | WWOX EGFR | head and neck carcinoma (Liu et al) colorectal cancer ( El-Daly et al) | Liu CJ, et al. miR-134 induces oncogenicity and metastasis in head and neck carcinoma through targeting WWOX gene. *Int J Cancer* 2014;134:811-821. | El-Daly SM, et al. miRs-134 and -370 function as tumor suppressors in colorectal cancer by independently suppressing EGFR and PI3K signalling. *Sci Rep* 2016;6:24720. | - |  |
| miR-140-5p | ○ | ○ |  | 1.7814485 | 2.3004813 | 2.2831893 |  | 1.6356138 | 1.9255729 | 1.7080433 | 1.939058183 | chemoresistance invasion inhibition | HDAC4 SMAD2 | osteosarcoma (Song et al) colorectal cancer (Zhai et al) | Song B, et al. Mechanism of chemoresistance mediated by miR-140 in human osteosarcoma and colon cancer cells. *Oncogene* 2009;28:4065-4074. | Zhai H, et al. Inhibition of colorectal cancer stem cell survival and invasive potential by -miR-140-5p mediated suppression of Smad2 and autophagy. *Oncotarget* 2015;6:19735-19746. | - |  |
| miR-150-5p | ○ | - |  | 19.909971 | 6.0215073 | 2.6838615 |  | 20.23272 | 4.722426 | 1.5921004 | 9.193764367 | senescence induction cell growth inhibition metastasis inhibition | DKC1 AKT2 FLT3 MYB CCR6 | NK/T cell lymphoma (Watanabe et al) MLL-associated leukemia (Jiang et al) cutaneous T-cell lymphoma (Ito et al) | Watanabe A, et al. The role of microRNA-150 as a tumor suppressor in malignant lymphoma. *Leukemia* 2011;25:1324-1334. | Jiang X, et al. Blockade of miR-150 maturation by MLL-fusion/MYC/LIN-28 is required for MLL-associated leukemia. *Cancer Cell* 2012;22:524-535. | Ito M, et al. MicroRNA-150 inhibits tumor invasion and metastasis by targeting the chemokine receptor CCR6, in advanced cutaneous T-cell lymphoma. *Blood* 2014;123:1499-1511. |  |
| miR-181a-5p | ○ | ○ |  | 2.7595158 | 3.3859565 | 3.4883103 |  | 2.6380374 | 3.4391103 | 2.5477405 | 3.0431118 | apoptosis induction anti-apoptosis migration invasion | GRP78 BIM SMAD7 | prostate cancer (Su et al) breast cancer (Taylor et al) ovarian cancer (Parikh et al) | Su SF, et al. miR-30d, miR-181a and miR-199a-5p cooperatively suppress the endoplasmic reticulum chaperone and signaling regulator GRP78 in cancer. *Oncogene* 2013;32:4694-4701. | Taylor MA, et al. TGF-β upregulates miR-181a expression to promote breast cancer metastasis. *J Clin Invest* 2013;123:150-163. | Parikh A, et al. microRNA-181a has a critical role in ovarian cancer progression through the regulation of the epithelial-mesenchymal transition. *Nat Commun* 2014;5:2977. |  |
| miR-181b-5p | ○ | ○ |  | 2.6265073 | 3.2539182 | 2.6682928 |  | 2.3747702 | 3.0951917 | 1.5410924 | 2.593295433 | cell growth apoptosis induction cell cycle progression | TIMP3 MCL1 RASSF1A | hepatocellular carcinoma (Wang et al) chronic lymphocytic leukemia (Visone et al) acute promyelocytic Leukemia (Bräuer et al) | Wang B, et al. TGFbeta-mediated upregulation of hepatic miR-181b promotes hepatocarcinogenesis by targeting TIMP3. *Oncogene* 2010;29:1787-1797. | Visone R, et al. miR-181b is a biomarker of disease progression in chronic lymphocytic leukemia. *Blood* 2011;118:3072-3079. | Bräuer-Hartmann D, et al. MV2PML/RARα-Regulated miR-181a/b Cluster Targets the Tumor Suppressor RASSF1A in Acute Promyelocytic Leukemia. *Cancer Res* 2015;75:3411-3424. |  |
| miR-181c-5p | - | ○ |  | 22.9585 | 4.125654 | 3.1869552 |  | 21.051193 | 3.6098366 | 2.659336 | 9.598579133 | brain metastasis chemoresistance | PDPK1 MST1 LATS2 | breast cancer (Tominaga et al) pancreatic cancer (Chen et al) | Tominaga N, et al. Brain metastatic cancer cells release microRNA-181c-containing extracellular vesicles capable of destructing blood-brain barrier. *Nat Commun* 2015;6:6716. | Chen M, et al. Upregulation of miR-181c contributes to chemoresistance in pancreatic cancer by inactivating the Hippo signaling pathway. *Oncotarget* 2015;6:44466-44479. | - |  |
| miR-181d | ○ | - |  | 6.4855776 | 1.9258054 | 5.1895456 |  | 2.3774445 | 1.8350776 | 3.57356 | 3.564501783 | chemosensitivity | MGMT | glioblastoma (Khalil et al) | Khalil S, et al. miRNA array screening reveals cooperative MGMT-regulation between miR-181d-5p and miR-409-3p in glioblastoma. *Oncotarget* 2016:Epub ahead of print. | - | - |  |
| miR-183-5p | ○ | ○ |  | 40.093246 | 129.87228 | 6.485477 |  | 24.7301 | 122.0301 | 7.4630456 | 55.11237477 | migraton apoptoss induction metastasis inhibition | EGR1 FOXF2 | synovial sarcoma (Sarver et al) neuroblastoma (Londrini et al) lung cancer (Kundu et al) | Sarver AL, et al. MicroRNA miR-183 functions as an oncogene by targeting the transcription factor EGR1 and promoting tumor cell migration. *Cancer Res* 2010;70:9570-9580. | Lodrini M, et al. MYCN and HDAC2 cooperate to repress miR-183 signaling in neuroblastoma. *Nucleic Acids Res* 2013;41:6018-6033. | Kundu ST, et al. The miR-200 family and the miR-183~96~182 cluster target Foxf2 to inhibit invasion and metastasis in lung cancers. *Oncogene* 2016;35:173-186. |  |
| miR-185-5p | ○ | - |  | 2.229965 | 2.432709 | 2.4319825 |  | 1.8714561 | 2.0971332 | 2.0431051 | 2.184391817 | cell growth inhibition chemosensitivity metastasis inhibition | SIX1 DNMT1 STIM1 | ovarian cancer (Imam et al, Xiang et al) colorectal cancer (Zhang et al) | Imam JS, et al. MicroRNA-185 suppresses tumor growth and progression by targeting the Six1 oncogene in human cancers. *Oncogene* 2010;29:4971-4979. | Xiang Y, et al. MiR-152 and miR-185 co-contribute to ovarian cancer cells cisplatin sensitivity by targeting DNMT1 directly: a novel epigenetic therapy independent of decitabine. *Oncogene* 2014;33:378-386. | Zhang Z, et al. STIM1, a direct target of microRNA-185, promotes tumor metastasis and is associated with poor prognosis in colorectal cancer. *Oncogene* 2015;34:4808-4820. |  |
| miR-186-5p | ○ | - |  | 2.374095 | 3.4069023 | 2.719791 |  | 2.1102762 | 3.6166775 | 1.6914511 | 2.65319885 | cell cycle arrest chemosensitivity apoptosis induction | CCND1 TWIST1 XIAP PAK7 | lung cancer (Cai et al) ovarian cancer (Zhu et al) glioma (Zheng et al) | Cai J, et al. miR-186 downregulation correlates with poor survival in lung adenocarcinoma, where it interferes with cell-cycle regulation. *Cancer Res* 2013;73:756-766. | Zhu X, et al. miR-186 regulation of Twist1 and ovarian cancer sensitivity to cisplatin. *Oncogene* 2016;35:323-332. | Zheng J, et al. CRNDE affects the malignant biological characteristics of human glioma stem cells by negatively regulating miR-186. *Oncotarget* 2015;6:25339-25555. |  |
| miR-188-5p | ○ | - |  | 88.2318 | 10.842705 | 8.675543 |  | 73.126175 | 13.653568 | 6.0574317 | 33.43120378 | cell growth inhibition | LAPTM4B | prostate cancer (Zhang et al) | Zhang H, et al. miR-188-5p inhibits tumour growth and metastasis in prostate cancer by repressing LAPTM4B expression. *Oncotarget* 2015;6:6092-6104. | - | - |  |
| miR-192-5p | ○ | - |  | 7.549404 | 183.35388 | 16.882605 |  | 6.181579 | 184.93008 | 23.012356 | 70.31831733 | cell cycle arrest metastasis inhibition anti-angiogenesis | CDC7 MDM2 EGR1 | colon cancer (Georges et al) multiple myeloma (Pichiorri et al) ovarian cancer (Wu et al) | Georges SA, et al. Coordinated regulation of cell cycle transcripts by p53-Inducible microRNAs, miR-192 and miR-215. *Cancer Res* 2008;68:10105-10112. | Pichiorri F, et al. Downregulation of p53-inducible microRNAs 192, 194, and 215 impairs the p53/MDM2 autoregulatory loop in multiple myeloma development. *Cancer Cell* 2010;18:367-81. | Wu SY, et al. A miR-192-EGR1-HOXB9 regulatory network controls the angiogenic switch in cancer. *Nat Commun* 2016;7:11169. |  |
| miR-194-5p | ○ | - |  | 11.104322 | 74.98117 | 14.226597 |  | 8.799395 | 83.45342 | 20.12454 | 35.44824067 | invasion inhibition cell growth inhibition metastasis inhibition | BMI1 MDM2 BMP1 | endometrial cancer (Dong et al) multiple myeloma (Pichiorri et al)  non-small cell lung cancer (Wu et al) | Dong P, et al. MicroRNA-194 inhibits epithelial to mesenchymal transition of endometrial cancer cells by targeting oncogene BMI-1. *Mol Cancer* 2011;10:99. | Pichiorri F, et al. Downregulation of p53-inducible microRNAs 192, 194, and 215 impairs the p53/MDM2 autoregulatory loop in multiple myeloma development. *Cancer Cell* 2010;18:367-82. | Wu X, et al. miR-194 suppresses metastasis of non-small cell lung cancer through regulating expression of BMP1 and p27(kip1). *Oncogene* 2014;33:1506-1514. |  |
| miR-197-3p | ○ | - |  | 4.442778 | 2.5570068 | 2.5913818 |  | 2.7490008 | 1.7450191 | 1.829699 | 2.652480917 | cell growth inhibiton apoptosis induction | FUS1 MCL1 | lung cancer (Du et al) multiple meyloma (Yang et al) | Du L, et al. miR-93, miR-98, and miR-197 regulate expression of tumor suppressor gene FUS1. *Mol Cancer Res* 2009;7:1234-1243. | Yang Y, et al. miR-137 and miR-197 Induce Apoptosis and Suppress Tumorigenicity by Targeting MCL-1 in Multiple Myeloma. *Clin Cancer Res* 2015;21:2399-2411. | - |  |
| miR-197-5p | - | - |  | 2.98458 | 2.2678235 | 6.287096 |  | 2.2599697 | 2.6594872 | 4.4835925 | 3.490424817 | - | - | - | - | - | - |  |
| miR-210 | ○ | ○ |  | 3.2233248 | 4.839564 | 2.0968435 |  | 2.882008 | 2.7653663 | 1.8433335 | 2.941740017 | hypoxic response T-cell suppression | SHIP1 IL16 CXCL12 | myelodisplastic syndrome (Lee et al) melanoma (Noman et al) | Lee DW, et al. Loss of SHIP-1 protein expression in high-risk myelodysplastic syndromes is associated with miR-210 and miR-155. *Oncogene* 2012;31:4085-4094. | Noman MZ, et al. Tumor-Promoting Effects of Myeloid-Derived Suppressor Cells Are Potentiated by Hypoxia-Induced Expression of miR-210. *Cancer Res* 2015;75:3771-3787. | - |  |
| miR-215 | ○ | - |  | 14.954772 | 67.675095 | 158.12892 |  | 11.342874 | 68.4598 | 206.28896 | 87.8084035 | cell cycle arrest | MDM2 | colon cancer (Braun et al) multiple myeloma (Pichiorri et al) | Braun CJ, et al. p53-Responsive micrornas 192 and 215 are capable of inducing cell cycle arrest. *Cancer Res* 2008;68:10094-10104. | Pichiorri F, et al. Downregulation of p53-inducible microRNAs 192, 194, and 215 impairs the p53/MDM2 autoregulatory loop in multiple myeloma development. *Cancer Cell* 2010;18:367-82. | - |  |
| miR-301a-3p | - | ○ |  | 2.4542892 | 3.3819618 | 3.1306775 |  | 2.051267 | 3.5017629 | 2.3924444 | 2.8187338 | migration invasion | NF-κB TGFBR2 SMAD4 | pancreatic adenocarcinoma (Lu Z et al)  colorectal cancer (Zhang W et al)  pancreatic ductal adenocarcinoma (Xia et al) | Lu Z, et al. miR-301a as an NF-κB activator in pancreatic cancer cells. *EMBO J* 2011;30:57-67. | Zhang W, et al. MicroRNA-301a promotes migration and invasion by targeting TGFBR2 in human colorectal cancer. *J Exp Clin Cancer Res* 2014;33:113. | Xia X, et al. MicroRNA-301a-3p promotes pancreatic cancer progression via negative regulation of SMAD4. *Oncotarget* 2015;6:21046-21063. |  |
| miR-301b | - | - |  | 3.0021632 | 4.481835 | 3.1119082 |  | 2.680099 | 4.904815 | 2.4102685 | 3.43184815 | - | - | - | - | - | - |  |
| miR-320a | ○ | - |  | 2.7172265 | 3.2171557 | 1.9898896 |  | 2.582509 | 2.6105938 | 1.5649742 | 2.447058133 | cell growth inhibition migration inhibition | ETS2 SOX4 FOXM1 FOXQ1 | stromal cells (Bronisz et al) colorectal cancer (Vishnubalaji et al) | Bronisz A, et al. Reprogramming of the tumour microenvironment by stromal PTEN-regulated miR-320. *Nat Cell Biol* 2011;14:159-167. | Vishnubalaji R, et al. MicroRNA-320 suppresses colorectal cancer by targeting SOX4, FOXM1, and FOXQ1. *Oncotarget* 2016;Epub ahead of print. | - |  |
| miR-320b | ○ | - |  | 2.459392 | 3.7129202 | 2.0326376 |  | 2.3794646 | 3.0886042 | 1.5218382 | 2.532476133 | cell growth inhibition | MYC | colorectal cancer (Wang et al) | Wang H, et al. miR-320b suppresses cell proliferation by targeting c-Myc in human colorectal cancer cells. *BMC Cancer* 2015;15:748. | - | - |  |
| miR-320c | ○ | - |  | 3.0520303 | 8.055545 | 4.0611296 |  | 2.806562 | 10.199251 | 3.147866 | 5.220397317 | cell growth inhibition | CDK6 | bladder cancer (Wang et al) | Wang X, et al. MicroRNA-320c inhibits tumorous behaviors of bladder cancer by targeting Cyclin-dependent kinase 6. *J Exp Clin* *Cancer Res* 2014;33:69 | - | - |  |
| miR-320d | ○ | - |  | 2.8873787 | 4.0981674 | 2.9220972 |  | 2.6737742 | 3.6622474 | 2.3368738 | 3.09675645 | cell growth inhibition | SOX4 FOXM1 FOXQ1 | colorectal cancer (Vishnubalaji et al) | Vishnubalaji R, et al. MicroRNA-320 suppresses colorectal cancer by targeting SOX4, FOXM1, and FOXQ1. *Oncotarget* 2016;Epub ahead of print. | - | - |  |
| miR-320e | ○ | - |  | 2.96685 | 4.2197623 | 3.0046427 |  | 2.6892061 | 3.8584423 | 2.091742 | 3.1384409 | cell growth inhibition | SOX4 FOXM1 FOXQ2 | colorectal cancer (Vishnubalaji et al) | Vishnubalaji R, et al. MicroRNA-320 suppresses colorectal cancer by targeting SOX4, FOXM1, and FOXQ1. *Oncotarget* 2016;Epub ahead of print. | - | - |  |
| miR-371a-5p | ○ | - |  | 13.1732 | 4.452295 | 3.8832636 |  | 9.36618 | 2.8482003 | 3.499069 | 6.203701317 | stemness inhibition metastasis inhibition | SOX2 | colorectal cancer (Li et al) | Li Y, et al. The SOX17/miR-371-5p/SOX2 axis inhibits EMT, stem cell properties and metastasis in colorectal cancer. *Oncotarget* 2015;6:9099-9112. | - | - |  |
| miR-371b-5p | - | - |  | 13.141773 | 19.198692 | 12.580263 |  | 8.71935 | 29.265636 | 9.93711 | 15.473804 | - | - | - | - | - | - |  |
| miR-425-5p | - | ○ |  | 2.4892359 | 2.8676243 | 3.1825743 |  | 2.1783528 | 2.5165322 | 2.6326745 | 2.644499 | cell growth migration invasion | CTNNA3 | hepatocellular carcinoma (He et al) | He B, et al. CTNNA3 is a tumor suppressor in hepatocellular carcinomas and is inhibited by miR-425. *Oncotarget* 2016;7:8078-8089. | - | - |  |
| miR-449a | ○ | - |  | 18.613832 | 224.34016 | 46.5736 |  | 15.555946 | 229.12576 | 78.384094 | 102.0988987 | cell growth inhibition　　　　 migration inhibition | MET CCNE2 SIRT1 CDK6 | gastric cancer (Bou Kheir et al)  non-small cell lung cancer (Luo et al) | Bou Kheir T,et al. miR-449 inhibits cell proliferation and is down-regulated in gastric cancer. *Mol Cancer* 2011;10:29. | Luo W, et al. MicroRNA-449a is downregulated in non-small cell lung cancer and inhibits migration and invasion by targeting c-Met. *PLoS One* 2013;8:e64759. | - |  |
| miR-454-3p | - | ○ |  | 2.4638526 | 3.1264822 | 4.680705 |  | 2.1678739 | 3.4546068 | 3.3854284 | 3.21315815 | cell growth invasion | CHD5 | hepatocellular carcinoma (Yu et al) | Yu L et al. miR-454 functions as an oncogene by inhibiting CHD5 in hepatocellular carcinoma. *Oncotarget* 2015;6:39225-39234. | - | - |  |
| miR-483-5p | ○ | ○ |  | 13.070521 | 8.570265 | 8.536621 |  | 7.65204 | 15.367726 | 6.0848985 | 9.88034525 | cell growth inhibition invasion metastasis | ERK1 ALCAM FIS1 | glioma (Wang et al) lung adenocarcinoma (Song et al) tongue squamous cell carcinoma (Fan et al) | Wang L et al. MiR-483-5p suppresses the proliferation of glioma cells via directly targeting ERK1. *FEBS Lett* 2012;586:1312-1317. | Song Q et al. miR-483-5p promotes invasion and metastasis of lung adenocarcinoma by targeting RhoGDI1 and ALCAM. *Cancer Res* 2014;74:3031-3042. | Fan S et al. miR-483-5p determines mitochondrial fission and cisplatin sensitivity in tongue squamous cell carcinoma by targeting FIS1. *Cancer Lett* 2015;362:183-191. |  |
| miR-572 | - | ○ |  | 5.988269 | 6.4535546 | 70.24019 |  | 4.223188 | 9.429465 | 61.897896 | 26.37209377 | cell growth cell cycle progression | SOCS1 CDKN1A | ovarian cancer (Zhang et al) | Zhang X et al. Upregulation of miR-572 transcriptionally suppresses SOCS1 and p21 and contributes to human ovarian cancer progression. *Oncotarget* 2015;6:15180-15193. | - | - |  |
| miR-574-5p | ○ | ○ |  | 4.737685 | 3.7930346 | 10.509915 |  | 3.1085188 | 4.6961966 | 8.14651 | 5.831976667 | cell growth inhibition | CERS1 FOXN3 | lung cancer (Li et al) | Meyers-Needham M et al. Concerted functions of HDAC1 and microRNA-574-5p repress alternatively spliced ceramide synthase 1 expression in human cancer cells. *EMBO Mol Med* 2012;4:78-92. | Li Q et al. MicroRNA-574-5p was pivotal for TLR9 signaling enhanced tumor progression via down-regulating checkpoint suppressor 1 in human lung cancer. *PLoS One* 2012;7:e48278. | - |  |
| miR-590-5p | - | ○ |  | 2.072618 | 2.057338 | 2.2184505 |  | 1.7258551 | 1.9335649 | 1.576157 | 1.930663917 | proliferation invasion | RB1 | T-lymphoblastic leukaemia (Miao et al) | Miao MH et al. miR-590 promotes cell proliferation and invasion in T-cell acute lymphoblastic leukaemia by inhibiting RB1. *Oncotarget* 2016;Epub ahead of print. | - | - |  |
| miR-630 | ○ | - |  | 4.1830816 | 12.708733 | 7.530183 |  | 4.2674303 | 19.834751 | 6.9315414 | 9.24262005 | apoptosis induction cell growth inhibition | IGF1R MTDH | pancreatic cancer (Farhana et al) breast cancer (Corcoran et al)  breast cancer (Zhou et al) | Farhana L et al. Upregulation of miR-150* and miR-630 induces apoptosis in pancreatic cancer cells by targeting IGF-1R. *PLoS One* 2013;8:e61015. | Corcoran C et al. miR-630 targets IGF1R to regulate response to HER-targeting drugs and overall cancer cell progression in HER2 over-expressing breast cancer. *Mol Cancer* 2014;13:71. | Zhou CX. MiR-630 suppresses breast cancer progression by targeting metadherin. *Oncotarget* 2016;7:1288-1299. |  |
| miR-638 | ○ | ○ |  | 5.250428 | 6.296506 | 5.8914056 |  | 4.062973 | 9.195599 | 5.054882 | 5.958632267 | invasion inhibition  differentiation  cell growth inhibition cell growth  invasion | SOX2 p53 PTEN BRCA CDK2 | colorectal carcinoma (Ma et al) colon cancer and osteosarcoma (Tay et al) colorectal carcinoma (Zhang et al) | Ma K et al. Loss of miR-638 in vitro promotes cell invasion and a mesenchymal-like transition by influencing SOX2 expression in colorectal carcinoma cells. *Mol Cancer* 2014;13:118. | Tay Y et al. Characterization of dual PTEN and p53-targeting microRNAs identifies microRNA-638/Dnm2 as a two-hit oncogenic locus. *Cell Rep* 2014;8:714-722. | Zhang J et al. MicroRNA-638 inhibits cell proliferation, invasion and regulates cell cycle by targeting tetraspanin 1 in human colorectal carcinoma. *Oncotarget* 2014;5:12083-12096. |  |
| miR-642a-3p | - | - |  | 5.244507 | 9.468341 | 4.363682 |  | 4.1918716 | 12.076452 | 2.9705396 | 6.385898867 | - | - | - | - | - | - |  |
| miR-642b-3p | - | - |  | 5.651249 | 6.2489386 | 3.7295249 |  | 4.5592756 | 7.777428 | 2.6477757 | 5.1023653 | - | - | - | - | - | - |  |
| miR-663a | ○ | ○ |  | 5.049264 | 5.2737966 | 2.101618 |  | 5.18414 | 7.555943 | 1.6596311 | 4.470732117 | cell growth invasion inhibition | CDKN1A HSPG2 PIK3CD | nasopharyngeal carcinoma (Yi et al) breast cancer (Hu et al) glioblastoma (Shi et al) | Yi C et al. MiR-663, a microRNA targeting p21(WAF1/CIP1), promotes the proliferation and tumorigenesis of nasopharyngeal carcinoma. *Oncogene* 2012;31:4421-4433. | Hu H et al. The overexpression of hypomethylated miR-663 induces chemotherapy resistance in human breast cancer cells by targeting heparin sulfate proteoglycan 2 (HSPG2). *J Biol Chem* 2013;288:10973-10985. | Shi Y et al. Primate-specific miR-663 functions as a tumor suppressor by targeting PIK3CD and predicts the prognosis of human glioblastoma. *Clin Cancer Res* 2014;20:1803-1813. |  |
| miR-671-5p | - | - |  | 100.43982 | 100.193794 | 120.52072 |  | 73.73122 | 123.23548 | 109.852486 | 104.6622533 | - | - | - | - | - | - |  |
| miR-760 | - | - |  | 20.1829 | 10.8115 | 29.241898 |  | 10.067101 | 11.269198 | 14.773199 | 16.05763267 | - | - | - | - | - | - |  |
| miR-762 | - | ○ |  | 8.317445 | 8.871429 | 6.6473994 |  | 6.519171 | 7.541603 | 5.1865234 | 7.180595133 | cell growth invasion | IRF7 | breast cancer (Li et al) | Li Y et al. microRNA-762 promotes breast cancer cell proliferation and invasion by targeting IRF7 expression. *Cell Prolif* 2015;48:643-649. | - | - |  |
| miR-765 | ○ | - |  | 12.059678 | 5.4743285 | 8.997621 |  | 7.0009465 | 11.344562 | 4.855809 | 8.288824167 | cell growth inhibition migration inhibition | HMGA1 | prostate cancer (Leung et al) | Leung YK, et al. Hsa-miRNA-765 as a key mediator for inhibiting growth, migration and invasion in fulvestrant-treated prostate cancer. *PLoS One* 2014;9:e98037. | - | - |  |
| miR-769-5p | - | - |  | 4.9485493 | 8.122917 | 5.3170524 |  | 4.1947546 | 6.448122 | 4.2865787 | 5.552995667 | - | - | - | - | - | - |  |
| miR-874 | ○ | - |  | 6.393624 | 4.5954285 | 4.4280705 |  | 5.254355 | 3.8508651 | 2.6702406 | 4.532097283 | cell growth inhibition invasion inhibition  angiogenesis inhibition | PPP1CA HDAC1 STAT3 | squamous cell carcinoma (Nohata et al) head and neck carcinoma (Nohata et al) gastric cancer (Zhang et al) | Nohata N et al. Tumour suppressive microRNA-874 regulates novel cancer networks in maxillary sinus squamous cell carcinoma. *Br J Cancer* 2011;105:833-841. | Nohata N et al. Tumour-suppressive microRNA-874 contributes to cell proliferation through targeting of histone deacetylase 1 in head and neck squamous cell carcinoma. *Br J Cancer* 2013;108:1648-1658. | Zhang X et al. miR-874 functions as a tumor suppressor by inhibiting angiogenesis through STAT3/VEGF-A pathway in gastric cancer. *Oncotarget* 2015;6:1605-1617. |  |
| miR-937-5p | - | - |  | 10.625611 | 14.523773 | 9.375628 |  | 8.532787 | 8.2102 | 6.959689 | 9.704614667 | - | - | - | - | - | - |  |
| miR-939-5p | - | - |  | 2.8727973 | 2.4491322 | 3.6264637 |  | 2.409119 | 3.0459669 | 2.7775393 | 2.863503067 | - | - | - | - | - | - |  |
| miR-940 | ○ | ○ |  | 2.5670187 | 2.936005 | 2.0300698 |  | 2.3775902 | 2.134841 | 1.5837945 | 2.2715532 | migration inhibition invasion metastasis | MIEN1 ZNF24 | prostate cancer (Rajendiran et al) gastric cancer (Liu et al) | Rajendiran S et al. MicroRNA-940 suppresses prostate cancer migration and invasion by regulating MIEN1. *Mol Cancer* 2014;13:250. | Liu X et al. MicroRNA-940 promotes tumor cell invasion and metastasis by downregulating ZNF24 in gastric cancer. *Oncotarget* 2015;6:25418-25428. | - |  |
| miR-1202 | - | - |  | 4.1976795 | 9.574602 | 5.5521603 |  | 3.3837638 | 14.22324 | 3.4877431 | 6.73653145 | - | - | - | - | - | - |  |
| miR-1207-5p | ◯ | - |  | 5.8454657 | 11.984998 | 6.1968775 |  | 4.712274 | 15.946221 | 4.2371855 | 8.15383695 | cancer stem cell-like traits | SFRP1 | ovarian cancer (Wu et al) | Wu G, et al. MiR-1207 overexpression promotes cancer stem cell-like traits in ovarian cancer by activating the Wnt/β-catenin signaling pathway. *Oncotarget* 2015;6:28882-28894. | - | - |  |
| miR-1224-5p | - | - |  | 5.7324305 | 3.1224496 | 82.17318 |  | 5.412843 | 2.6356719 | 58.16901 | 26.2075975 | - | - | - | - | - | - |  |
| miR-1225-5p | - | - |  | 5.186486 | 10.765214 | 5.6121497 |  | 4.42306 | 15.522734 | 4.385951 | 7.649265783 | - | - | - | - | - | - |  |
| miR-1227-5p | - | - |  | 140.23262 | 125.995316 | 117.35262 |  | 89.8052 | 199.60289 | 91.06359 | 127.3420393 | - | - | - | - | - | - |  |
| miR-1229-5p | ◯ | - |  | 5.5590467 | 11.489225 | 4.4427347 |  | 4.8830075 | 18.324053 | 3.4042199 | 8.0170478 | cell growth | APC GSK3β ICAT | breast cancer (Tan et al) | Tan Z, et al. MicroRNA-1229 overexpression promotes cell proliferation and tumorigenicity and activates Wnt/β-catenin signaling in breast cancer. *Oncotarget* 2016;Epub ahead of print. | - | - |  |
| miR-1234-5p | - | ◯ |  | 5.970342 | 15.952566 | 7.044083 |  | 5.4684477 | 16.93123 | 5.2278514 | 9.432420017 | tumorigenesis | STAT3 | B cell lymphoma (Högfeldt et al) | Högfeldt T, et al. Expression of microRNA-1234 related signal transducer and activator of transcription 3 in patients with diffuse large B-cell lymphoma of activated B-cell like type from high and low infectious disease areas. *Leuk Lymphoma* 2014;55:1158-1165. | - | - |  |
| miR-1268a | - | - |  | 3.8555453 | 2.6545372 | 3.7367015 |  | 2.754345 | 3.2550106 | 2.5991645 | 3.142550683 | - | - | - | - | - | - |  |
| miR-1268b | - | - |  | 5.438733 | 3.099937 | 4.421588 |  | 3.7406273 | 4.689089 | 3.2397625 | 4.104956133 | - | - | - | - | - | - |  |
| miR-1275 | ◯ | - |  | 1.6711783 | 4.13434 | 1.8662043 |  | 1.6189868 | 8.055395 | 1.7227112 | 3.178135933 | cell growth inhibition | IGF2 | hepatocellular carcinoma (Fawzy et al) | Fawzy IO, et al. miR-1275: A single microRNA that targets the three IGF2-mRNA-binding proteins hindering tumor growth in hepatocellular carcinoma. *FEBS Lett* 2015;589:2257-2265. | - | - |  |
| miR-1290 | ◯ | - |  | 4.303506 | 2.8165448 | 5.258351 |  | 3.3278146 | 3.2092173 | 3.6912982 | 3.76778865 | - | KIF13B | colon cancer (Wu et al) | Wu J, et al. Up-regulation of microRNA-1290 impairs cytokinesis and affects the reprogramming of colon cancer cells. *Cancer Lett* 2013;329:155-163. | - | - |  |
| miR-1587 | - | - |  | 9.784496 | 9.523236 | 11.325675 |  | 6.458568 | 11.777185 | 8.18042 | 9.508263333 | - | - | - | - | - | - |  |
| miR-1915-3p | - | - |  | 8.263824 | 11.276943 | 8.141251 |  | 6.6164618 | 12.55784 | 7.163482 | 9.0033003 | - | - | - | - | - | - |  |
| miR-1973 | - | - |  | 6.655142 | 3.247616 | 3.1254108 |  | 6.3099623 | 4.556691 | 2.4700997 | 4.394153633 | - | - | - | - | - | - |  |
| miR-2392 | - | - |  | 12.866207 | 6.7795186 | 4.733583 |  | 7.903418 | 10.27701 | 3.758167 | 7.7196506 | - | - | - | - | - | - |  |
| miR-2861 | - | - |  | 7.735915 | 9.210257 | 7.8431497 |  | 5.613762 | 13.318747 | 6.8602433 | 8.430345667 | - | - | - | - | - | - |  |
| miR-3127-5p | - | ◯ |  | 2.271518 | 3.6676207 | 4.133844 |  | 2.0673156 | 1.7688731 | 2.9593372 | 2.8114181 | cell growth | PHLPP1/2 ABL1 | hepatocellular carcinoma (Jiang et al) lung cancer (Sun et al) | Jiang J, et al. MicroRNA-3127 promotes cell proliferation and tumorigenicity in hepatocellular carcinoma by disrupting of PI3K/AKT negative regulation. *Oncotarget* 2015;6:6359-6372. | Sun Y, et al. Reduced miR-3127-5p expression promotes NSCLC proliferation/invasion and contributes to dasatinib sensitivity via the c-Abl/Ras/ERK pathway. *Sci Rep* 2014;4:6527. | - |  |
| miR-3135b | - | - |  | 7.668401 | 5.9503193 | 8.542669 |  | 6.6716995 | 4.931408 | 5.5274305 | 6.54865455 | - | - | - | - | - | - |  |
| miR-3138 | - | - |  | 81.03079 | 70.0984 | 90.15187 |  | 51.36041 | 96.5788 | 68.80701 | 76.33788 | - | - | - | - | - | - |  |
| miR-3141 | - | - |  | 6.246307 | 3.212401 | 4.5937557 |  | 4.531982 | 4.0931787 | 3.4985797 | 4.362700683 | - | - | - | - | - | - |  |
| miR-3149 | - | - |  | 38.93909 | 71.5489 | 335.2739 |  | 15.2039995 | 60.72819 | 300.47995 | 137.0290049 | - | - | - | - | - | - |  |
| miR-3162-5p | - | - |  | 4.339457 | 2.6639018 | 2.6339645 |  | 3.1978326 | 2.3390884 | 1.9693061 | 2.8572584 | - | - | - | - | - | - |  |
| miR-3620-5p | - | - |  | 51.804806 | 40.412697 | 28.481195 |  | 33.7829 | 53.52138 | 24.382994 | 38.73099533 | - | - | - | - | - | - |  |
| miR-3652 | - | - |  | 99.55531 | 93.70732 | 41.564205 |  | 88.00308 | 96.279205 | 38.641994 | 76.29185233 | - | - | - | - | - | - |  |
| miR-3653 | - | - |  | 3.2961965 | 2.095923 | 2.1669436 |  | 3.1558383 | 2.7001462 | 1.6780205 | 2.51551135 | - | - | - | - | - | - |  |
| miR-3656 | - | - |  | 4.62245 | 5.4911423 | 4.6908717 |  | 3.8731782 | 6.8224487 | 3.715793 | 4.869313983 | - | - | - | - | - | - |  |
| miR-3663-3p | - | - |  | 19.304018 | 228.08449 | 51.542503 |  | 17.60689 | 224.89711 | 42.432896 | 97.31131783 | - | - | - | - | - | - |  |
| miR-3665 | - | - |  | 7.229107 | 7.8921413 | 5.498165 |  | 5.888682 | 5.841186 | 4.5842633 | 6.155590767 | - | - | - | - | - | - |  |
| miR-3679-5p | - | - |  | 6.4314933 | 7.04722 | 8.2661295 |  | 4.6366982 | 9.849752 | 6.56827 | 7.1332605 | - | - | - | - | - | - |  |
| miR-3940-5p | - | - |  | 9.777668 | 13.608203 | 50.65859 |  | 8.68506 | 8.617233 | 48.18669 | 23.255574 | - | - | - | - | - | - |  |
| miR-3960 | - | - |  | 6.0375876 | 11.6814375 | 4.0413804 |  | 5.3394523 | 11.384525 | 3.5372858 | 7.003611433 | - | - | - | - | - | - |  |
| miR-4257 | - | - |  | 114.690674 | 67.1332 | 6.02454 |  | 86.7405 | 56.06571 | 4.9428687 | 55.93291545 | - | - | - | - | - | - |  |
| miR-4270 | - | - |  | 5.491587 | 13.466982 | 6.5066104 |  | 4.8582053 | 19.44872 | 4.63373 | 9.067639117 | - | - | - | - | - | - |  |
| miR-4271 | - | - |  | 16.875458 | 165.49727 | 9.227735 |  | 11.744859 | 185.09677 | 7.7371783 | 66.02987838 | - | - | - | - | - | - |  |
| miR-4281 | - | - |  | 5.9315395 | 9.006747 | 5.569475 |  | 4.8241386 | 10.467556 | 3.970319 | 6.62829585 | - | - | - | - | - | - |  |
| miR-4298 | - | - |  | 2.5984545 | 2.1361625 | 3.2263901 |  | 2.1602252 | 2.9811177 | 2.473105 | 2.595909167 | - | - | - | - | - | - |  |
| miR-4299 | - | - |  | 5.948838 | 4.7418294 | 11.734824 |  | 3.7053916 | 5.1225986 | 8.275643 | 6.588187433 | - | - | - | - | - | - |  |
| miR-4306 | - | - |  | 2.61445 | 2.7664812 | 2.9835224 |  | 2.133378 | 2.7382858 | 1.9863203 | 2.53707295 | - | - | - | - | - | - |  |
| miR-4327 | - | - |  | 17.150438 | 147.48558 | 35.886898 |  | 15.106658 | 88.1251 | 29.794096 | 55.59146167 | - | - | - | - | - | - |  |
| miR-4419a | - | - |  | 30.0255 | 21.047396 | 30.292704 |  | 23.919998 | 24.767 | 25.079994 | 25.855432 | - | - | - | - | - | - |  |
| miR-4430 | - | - |  | 44.884007 | 63.313 | 50.68971 |  | 25.149597 | 55.242706 | 36.394196 | 45.945536 | - | - | - | - | - | - |  |
| miR-4433-3p | - | - |  | 34.21992 | 60.470394 | 6.3007646 |  | 22.694317 | 67.805084 | 5.5558105 | 32.84104835 | - | - | - | - | - | - |  |
| miR-4442 | - | - |  | 2.5767245 | 2.5723372 | 2.944922 |  | 2.0745754 | 3.262244 | 2.3421667 | 2.6288283 | - | - | - | - | - | - |  |
| miR-4455 | - | - |  | 138.18877 | 12.121092 | 14.334602 |  | 84.99738 | 11.456103 | 11.899169 | 45.49951933 | - | - | - | - | - | - |  |
| miR-4459 | - | - |  | 3.3361871 | 8.909871 | 3.2835572 |  | 3.451154 | 15.968539 | 3.4163463 | 6.394275767 | - | - | - | - | - | - |  |
| miR-4463 | - | - |  | 28.695137 | 114.362785 | 6.334177 |  | 18.064722 | 121.088425 | 5.844298 | 49.064924 | - | - | - | - | - | - |  |
| miR-4465 | - | - |  | 9.079073 | 23.110928 | 3.876887 |  | 7.9599085 | 6.5435605 | 3.272887 | 8.973874 | - | - | - | - | - | - |  |
| miR-4466 | - | - |  | 6.2926903 | 10.066329 | 5.597213 |  | 5.357356 | 8.340068 | 4.7506638 | 6.73405335 | - | - | - | - | - | - |  |
| miR-4481 | - | - |  | 7.380035 | 1.6934383 | 6.4119587 |  | 3.67761 | 3.124416 | 3.730038 | 4.336249333 | - | - | - | - | - | - |  |
| miR-4484 | - | - |  | 33.06802 | 5.99333 | 8.957849 |  | 23.916021 | 5.2828 | 7.8003335 | 14.16972558 | - | - | - | - | - | - |  |
| miR-4485 | - | - |  | 23.990862 | 6.5740504 | 14.587576 |  | 17.272379 | 7.4189024 | 11.7435465 | 13.59788605 | - | - | - | - | - | - |  |
| miR-4486 | - | - |  | 72.295105 | 53.756393 | 121.218 |  | 31.659796 | 58.613995 | 91.95771 | 71.58349983 | - | - | - | - | - | - |  |
| miR-4497 | - | - |  | 8.931481 | 6.4293847 | 17.708433 |  | 6.320431 | 5.365618 | 13.246598 | 9.66699095 | - | - | - | - | - | - |  |
| miR-4499 | - | - |  | 11.28255 | 4.7539115 | 3.963943 |  | 8.780496 | 5.967385 | 3.7134573 | 6.410290467 | - | - | - | - | - | - |  |
| miR-4505 | - | - |  | 8.20112 | 7.93756 | 8.590603 |  | 5.620009 | 10.450872 | 6.5656734 | 7.894306233 | - | - | - | - | - | - |  |
| miR-4507 | - | - |  | 9.055309 | 8.615253 | 10.708871 |  | 5.9781656 | 11.768179 | 8.184796 | 9.051762267 | - | - | - | - | - | - |  |
| miR-4516 | - | - |  | 5.182647 | 12.169146 | 3.2596653 |  | 4.914015 | 8.32719 | 3.1680346 | 6.170116317 | - | - | - | - | - | - |  |
| miR-4530 | - | - |  | 9.261639 | 12.094911 | 8.152221 |  | 7.687952 | 9.860319 | 6.92083 | 8.996312 | - | - | - | - | - | - |  |
| miR-4532 | - | - |  | 132.6881 | 97.71383 | 60.897793 |  | 96.17451 | 134.7288 | 55.353683 | 96.25945267 | - | - | - | - | - | - |  |
| miR-4534 | - | - |  | 114.72768 | 167.31291 | 112.04332 |  | 83.47118 | 252.84302 | 79.27128 | 134.9448983 | - | - | - | - | - | - |  |
| miR-4634 | - | - |  | 182.98814 | 174.56331 | 27.712395 |  | 158.95349 | 147.51839 | 34.455097 | 121.0318037 | - | - | - | - | - | - |  |
| miR-4669 | - | - |  | 4.714593 | 4.175513 | 4.8001895 |  | 3.5080173 | 5.118751 | 3.5643604 | 4.3135707 | - | - | - | - | - | - |  |
| miR-4672 | - | - |  | 7.850373 | 5.055414 | 7.37017 |  | 5.218929 | 7.9203935 | 6.2056766 | 6.603492683 | - | - | - | - | - | - |  |
| miR-4687-3p | - | - |  | 5.642241 | 9.656671 | 4.920595 |  | 5.0233207 | 8.483508 | 4.0636244 | 6.298326683 | - | - | - | - | - | - |  |
| miR-4690-5p | - | - |  | 85.6452 | 75.1187 | 121.94212 |  | 51.451595 | 84.74719 | 87.333916 | 84.37312017 | - | - | - | - | - | - |  |
| miR-4695-5p | - | - |  | 108.307 | 41.0818 | 23.546494 |  | 73.72288 | 37.871696 | 17.078175 | 50.2680075 | - | - | - | - | - | - |  |
| miR-4698 | - | - |  | 358.88104 | 64.8725 | 271.6321 |  | 241.80995 | 68.13019 | 215.67499 | 203.5001283 | - | - | - | - | - | - |  |
| miR-4701-3p | - | - |  | 8.56227 | 1.6667042 | 6.009054 |  | 3.4656148 | 2.1756988 | 4.655 | 4.4223903 | - | - | - | - | - | - |  |
| miR-4726-5p | - | - |  | 37.34949 | 132.61742 | 274.77103 |  | 15.884799 | 44.325592 | 227.23099 | 122.0298868 | - | - | - | - | - | - |  |
| miR-4739 | - | - |  | 7.812465 | 23.385147 | 7.2860885 |  | 7.741417 | 22.284876 | 7.109506 | 12.60324992 | - | - | - | - | - | - |  |
| miR-4741 | - | - |  | 7.1960354 | 10.8692255 | 3.317135 |  | 6.108267 | 15.05506 | 2.400745 | 7.491077983 | - | - | - | - | - | - |  |
| miR-4745-5p | - | - |  | 7.527289 | 6.2804303 | 33.071396 |  | 6.700353 | 4.685278 | 29.820696 | 14.68090705 | - | - | - | - | - | - |  |
| miR-4750-5p | - | - |  | 10.022599 | 1.6616749 | 6.784894 |  | 3.333144 | 1.6181531 | 4.9130077 | 4.72224545 | - | - | - | - | - | - |  |
| miR-4763-3p | - | - |  | 5.555084 | 12.676855 | 5.0173554 |  | 4.954047 | 8.786788 | 3.9033813 | 6.815585117 | - | - | - | - | - | - |  |
| miR-4778-5p | - | - |  | 6.968893 | 4.2762957 | 13.588923 |  | 4.38818 | 7.7876053 | 9.856101 | 7.810999667 | - | - | - | - | - | - |  |
| miR-4787-5p | - | - |  | 9.98846 | 10.990364 | 9.1894045 |  | 6.5506787 | 15.895788 | 7.350618 | 9.994218867 | - | - | - | - | - | - |  |
| miR-4788 | - | - |  | 4.0285945 | 4.4867873 | 4.997132 |  | 3.7868788 | 3.9028013 | 3.6712317 | 4.145570933 | - | - | - | - | - | - |  |
| miR-4800-5p | - | - |  | 3.8790529 | 3.4058602 | 3.535197 |  | 2.9964015 | 4.7795105 | 2.2836866 | 3.47995145 | - | - | - | - | - | - |  |
| miR-5001-5p | - | - |  | 9.042725 | 12.297925 | 10.817397 |  | 6.0976615 | 17.604702 | 9.457592 | 10.88633375 | - | - | - | - | - | - |  |
| miR-5006-5p | - | - |  | 5.2396064 | 3.5609944 | 2.3892684 |  | 3.841058 | 4.8290377 | 2.0785325 | 3.656416233 | - | - | - | - | - | - |  |
| miR-5194 | - | - |  | 2.7659926 | 4.414249 | 5.9805984 |  | 2.5264907 | 1.897925 | 4.4689293 | 3.6756975 | - | - | - | - | - | - |  |
| miR-5195-3p | - | - |  | 2.952071 | 2.90244 | 2.8582835 |  | 2.3735342 | 3.4228146 | 1.648583 | 2.692954383 | - | - | - | - | - | - |  |
| miR-5703 | - | - |  | 3.8662295 | 12.27164 | 6.5220923 |  | 4.2867594 | 20.38722 | 6.68302 | 9.002826867 | - | - | - | - | - | - |  |
| miR-5787 | ◯ | - |  | 6.7602887 | 22.839153 | 9.633434 |  | 5.4258976 | 33.68574 | 6.8589373 | 14.2005751 | cell growth inhibition | EIF5 | fibroblast (Yoo et al) | Yoo H, et al. The has-miR-5787 represses cellular growth by targeting eukaryotic translation initiation factor 5 (eIF5) in fibroblasts. *Biochem Biophys Res Commun* 2011;415:567-572. | - | - |  |
| miR-6068 | - | - |  | 8.614793 | 10.57548 | 9.987517 |  | 5.960755 | 16.816132 | 8.639835 | 10.09908533 | - | - | - | - | - | - |  |
| miR-6073 | - | - |  | 14.052629 | 65.532196 | 97.73062 |  | 9.10091 | 89.51759 | 73.121 | 58.17582417 | - | - | - | - | - | - |  |
| miR-6075 | - | - |  | 94.61252 | 87.29692 | 56.614796 |  | 52.1891 | 126.07396 | 46.645294 | 77.238765 | - | - | - | - | - | - |  |
| miR-6076 | - | - |  | 3.0848446 | 2.9136174 | 2.8683684 |  | 2.837028 | 2.9158669 | 2.31461 | 2.822389217 | - | - | - | - | - | - |  |
| miR-6086 | - | - |  | 44.881603 | 3.6936512 | 35.034603 |  | 41.618202 | 5.173654 | 28.143097 | 26.42413503 | - | - | - | - | - | - |  |
| miR-6087 | - | - |  | 4.7936788 | 7.035814 | 3.3040469 |  | 4.184383 | 8.210309 | 2.758262 | 5.04774895 | - | - | - | - | - | - |  |
| miR-6088 | - | - |  | 6.0351815 | 15.123691 | 5.5634317 |  | 6.0333085 | 15.364017 | 4.20908 | 8.721451617 | - | - | - | - | - | - |  |
| miR-6089 | - | - |  | 7.0640364 | 8.351065 | 5.0432224 |  | 5.740712 | 8.742524 | 4.411863 | 6.5589038 | - | - | - | - | - | - |  |
| miR-6090 | - | - |  | 7.5948334 | 16.057512 | 4.6369734 |  | 6.905489 | 9.651814 | 3.8276415 | 8.112377217 | - | - | - | - | - | - |  |
| miR-6124 | - | - |  | 5.6825824 | 4.7879577 | 4.4314594 |  | 4.3941007 | 6.4024544 | 3.449302 | 4.8579761 | - | - | - | - | - | - |  |
| miR-6125 | - | - |  | 5.9590535 | 6.9535627 | 6.136187 |  | 4.4590545 | 8.907436 | 5.1533566 | 6.261441717 | - | - | - | - | - | - |  |
| miR-6126 | - | - |  | 4.6844945 | 9.605078 | 4.7433486 |  | 4.2520404 | 5.433467 | 3.4515586 | 5.361664517 | - | - | - | - | - | - |  |
| miR-6130 | - | - |  | 36.325096 | 116.232475 | 252.811 |  | 32.965397 | 43.045704 | 224.73097 | 117.685107 | - | - | - | - | - | - |  |
| miR-6510-5p | - | - |  | 5.6777606 | 6.688788 | 6.1745305 |  | 4.315989 | 9.687957 | 4.6428976 | 6.197987117 | - | - | - | - | - | - |  |
| miR-6722-3p | - | - |  | 66.97208 | 46.572784 | 41.835392 |  | 54.264584 | 52.666786 | 36.453197 | 49.79413717 | - | - | - | - | - | - |  |
| miR-6724-5p | - | - |  | 4.3616376 | 5.200948 | 3.7859867 |  | 3.6049278 | 5.119183 | 2.7717485 | 4.1407386 | - | - | - | - | - | - |  |
