# Supplementary material for: Histone deacetylase inhibitors inhibit metastasis by restoring a tumor suppressive microRNA-150 in advanced cutaneous T-cell lymphoma
Source: Oncotarget. 2016 Dec 7;8(5):7572–85. doi: 10.18632/oncotarget.13810 (PMC5352344; doi:10.18632/oncotarget.13810)
Supplement: Supplementary file 3 [file oncotarget-08-7572-s003.docx]

| Table S2. Northern blot analysis for HDACis induced miRNA that possesses seed sequence of CCR6. | | |  |  |  |  |  |  |  |  |  |  |
| --- | --- | --- | --- | --- | --- | --- | --- | --- | --- | --- | --- | --- |
| miRNA name | Accession number(miR base) | Sequence of mature miRNAs(5'-) | Genome context (stem roop) | Fold change (average) | CD4-1 | CD4-2 | My-La | MJ | HH | HUT78 | CTCL1 (tumor) | CTCL2 (tumor) |
| hsa-miR-96-5p | MIMAT0000095 | 5’-UUUGGCACUAGCACAUUUUUGCU-3’ | chr7: 129774692-129774769 | 55.18 | - | - | - | - | - | - | - | - |
| hsa-miR-150-5p | MIMAT0000451 | 5’-UCUCCCAACCCUUGUACCAGUG-3’ | chr19: 49500785-49500868 | 12.69 | +++ | +++ | - | - | - | - | + | +/- |
| hsa-miR-183-5p | MIMAT0000261 | 5’-UAUGGCACUGGUAGAAUUCACU-3’ | chr7: 129774905-129775014 | 55.11 | - | - | - | - | - | - | - | - |
| hsa-miR-185-5p | MIMAT0000455 | 5'-UGGAGAGAAAGGCAGUUCCUGA-3' | chr22: 20033139-20033220 | 2.06 | ++ | ++ | +/- | +/- | +/- | +/- | +/- | +/- |
| hsa-miR-186-5p | MIMAT0000456 | 5'-CAAAGAAUUCUCCUUUUGGGCU-3' | chr1: 71067631-71067716 | 2.7 | - | - | - | - | - | - | - | - |
| hsa-miR-194-5p | MIMAT0000460 | 5’-UGUAACAGCAACUCCAUGUGGA-3’ | chr1: 220118157-220118241 | 35.5 | - | - | - | - | - | - | - | - |
| hsa-miR-301a-3p | MIMAT0000688 | 5'-CAGUGCAAUAGUAUUGUCAAAGC-3' | chr17: 59151136-59151221 | 2.84 | +/- | +/- | +/- | +/- | +/- | +/- | +/- | +/- |
| hsa-miR-301b | MIMAT0032026 | 5’-CAGUGCAAUGAUAUUGUCAAAGC-3’ | chr22: 21652981-21653058 | 3.77 | - | - | - | - | - | - | +/- | - |
| hsa-miR-320a | MIMAT0000510 | 5'-AAAAGCUGGGUUGAGAGGGCGA-3' | chr8: 22244962-22245043 | 2.53 | - | - | - | - | +/- | - | + | - |
| hsa-miR-320b | MIMAT0005792 | 5’-AAAAGCUGGGUUGAGAGGGCAA-3’ | chr1: 116671749-116671827 | 1.52 | +/- | +/- | - | - | - | - | + | +/- |
| hsa-miR-320c | MIMAT0005793 | 5’-AAAAGCUGGGUUGAGAGGGU-3’ | chr18: 21683510-21683597 | 5.35 | +/- | +/- | - | - | +/- | - | + | +/- |
| hsa-miR-320d | MIMAT0006764 | 5’-AAAAGCUGGGUUGAGAGGA-3’ | chr13: 40727828-40727875 | 2.56 | +/- | +/- | - | - | - | - | + | +/- |
| hsa-miR-371a-5p | MIMAT0004687 | 5’-ACUCAAACUGUGGGGGCACU-3’ | chr19: 53787675-53787741 | 5.17 | - | - | - | - | - | - | - | - |
| hsa-miR-371b-5p | MIMAT0019892 | 5’-ACUCAAAAGAUGGCGGCACUUU-3’ | chr19: 53787677-53787742 | 15.91 | - | - | - | - | - | - | - | - |
| hsa-miR-454-3p | MIMAT0003885 | 5'-UAGUGCAAUAUUGCUUAUAGGGU-3' | chr17: 59137758-59137872 | 3.39 | +/- | +/- | - | - | - | - | - | - |
| hsa-miR-937-5p | MIMAT0022938 | 5'-GUGAGUCAGGGUGGGGCUGG-3' | chr8: 143812957-143813042 | 9.83 | +/- | +/- | +/- | +/- | - | - | +++ | + |
| hsa-miR-939-5p | MIMAT0004982 | 5'-UGGGGAGCUGAGGCUCUGGGGGUG-3' | chr8: 144394149-144394230 | 2.85 | - | - | - | - | - | - | + | - |
| hsa-miR-1915-3p | MIMAT0007892 | 5'-CCCCAGGGCGACGCGGCGGG-3' | chr10: 21496562-21496641 | 9.37 | ND | ND | ND | ND | ND | ND | ND | ND |
| hsa-miR-3135b | MIMAT0018985 | 5’-GGCUGGAGCGAGUGCAGUGGUG-3’ | chr6: 32749912-32749979 | 6.73 | - | - | - | - | - | - | + | - |
| hsa-miR-3149 | MIMAT0015022 | 5'-UUUGUAUGGAUAUGUGUGUGUAU-3' | chr8: 76966768-76966850 | 137.03 | ND | ND | ND | ND | ND | ND | ND | ND |
| hsa-miR-3162-5p | MIMAT0015036 | 5'-UUAGGGAGUAGAAGGGUGGGGAG-3' | chr11: 59595077-59595158 | 2.58 | ND | ND | ND | ND | ND | ND | ND | ND |
| hsa-miR-3652 | MIMAT0018072 | 5’-CGGCUGGAGGUGUGAGGA-3’ | chr12: 103930425-103930555 | 76.29 | - | +/- | - | - | - | - | + | +/- |
| hsa-miR-3653 | MIMAT0032110 | 5'-CCUCCUGAUGAUUCUUCUUC-3' | chr22: 29333158-29333267 | 2.3 | - | - | - | - | - | - | +/- | - |
| hsa-miR-3663-3p | MIMAT0018085 | 5’-UGAGCACCACACAGGCCGGGCGC-3’ | chr10: 117167678-117167774 | 171.45 | ND | ND | ND | ND | ND | ND | ND | ND |
| hsa-miR-4306 | MIMAT0016858 | 5'-UGGAGAGAAAGGCAGUA-3' | chr13: 99643059-99643149 | 2.53 | +/- | +/- | - | - | - | - | +/- | - |
| hsa-miR-4430 | MIMAT0018945 | 5'-AGGCUGGAGUGAGCGGAG-3' | chr2: 33418516-33418564 | 45.95 | ND | ND | ND | ND | ND | ND | ND | ND |
| hsa-miR-4534 | MIMAT0019073 | 5’-GGAUGGAGGAGGGGUCU-3’ | chr22: 37988794-37988853 | 134.94 | - | - | - | - | - | - | + | - |
| hsa-miR-4687-3p | MIMAT0019775 | 5'-UGGCUGUUGGAGGGGGCAGGC-3' | chr11: 3856062-3856141 | 6.21 | ND | ND | ND | ND | ND | ND | ND | ND |
| hsa-miR-4698 | MIMAT0019793 | 5’-UCAAAAUGUAGAGGAAGACCCCA-3’ | chr12: 47187812-47187891 | 203.50 | - | - | - | - | - | - | - | - |
| hsa-miR-5006-5p | MIMAT0021033 | 5’-UUGCCAGGGCAGGAGGUGGAA-3’ | chr13: 41568286-41568395 | 3.05 | ND | ND | ND | ND | ND | ND | ND | ND |
| hsa-miR-5194 | MIMAT0021125 | 5'-UGAGGGGUUUGGAAUGGGAUGG-3' | chr8: 130008334-130008453 | 3.63 | ND | ND | ND | ND | ND | ND | ND | ND |
| hsa-miR-6088 | MIMAT0023713 | 5’-AGAGAUGAAGCGGGGGGGCG-3’ | chr19: 45436654-45436704 | 8.94 | +/- | +/- | +/- | +/- | +/- | +/- | +++ | + |
| hsa-miR-6124 | MIMAT0024597 | 5’-GGGAAAAGGAAGGGGGAGGA-3’ | chr11: 12163683-12163767 | 4.75 | - | - | - | - | - | - | ++ | - |
| hsa-miR-6126 | MIMAT0024599 | 5’-GUGAAGGCCCGGCGGAGA-3’ | chr16: 3485381-3485469 | 5.12 | - | +/- | +/- | +/- | + | +/- | ++ | + |
| hsa-miR-6724-5p | MIMAT0025856 | 5’-CUGGGCCCGCGGCGGGCGUGGGG-3’ | chr21: 8205315-8205406 | 4.05 | - | - | - | - | - | - | ++ | - |
